# Elimination of *Fusobacterium nucleatum* With Biomimetic Nanoparticles to Reverse Tumor Immunosuppression and Enhance Colorectal Cancer Therapy

**DOI:** 10.1002/EXP.20250551

**Published:** 2026-06-26

**Authors:** Mengting Zhou, Jiahao Du, Cheng Li, Yefei Zhu, Kairuo Wang, Keyi Wen, Yuanyuan Zhang, Sy‐Tsong Dean Chueng, Qian Chen, Yang Zhang, Huanlong Qin

**Affiliations:** ^1^ Nanotechnology and Intestinal Microecology Research Center, Shanghai Tenth People's Hospital, School of Medicine Tongji University Shanghai China; ^2^ Affiliated Suzhou Hospital of Nanjing Medical University, Suzhou Municipal Hospital, Gusu School Nanjing Medical University Suzhou China; ^3^ Department of Anesthesiology and Perioperative Medicine, Shanghai Key Laboratory of Anesthesiology and Brain Functional Modulation, Clinical Research Center for Anesthesiology and Perioperative Medicine, Translational Research Institute of Brain and Brain‐Like Intelligence, Shanghai Fourth People's Hospital, School of Medicine Tongji University Shanghai China; ^4^ Department of Gastroenterology The Second Affiliated Hospital of Xi'an Jiaotong University Xi'an Shaanxi China; ^5^ Department of Chemistry and Chemical Biology Rutgers The State University of New Jersey Piscataway New Jersey USA; ^6^ Department of Pharmacy Shanghai Eighth People's Hospital Shanghai China

**Keywords:** biomimetic nanoparticles, colorectal cancer, *Fusobacterium nucleatum*, sonodynamic therapy, tumor microenvironment

## Abstract

Tumor‐associated microbes like *Fusobacterium nucleatum* (*F. nucleatum*) critically contribute to immunosuppression and hinder cancer therapy. To overcome this challenge, we developed a microbe‐targeted immune reprogramming strategy utilizing tumor cell membrane‐camouflaged Ag@MSN‐PpIX nanoparticles (M‐MAP). Under ultrasound irradiation, M‐MAP specifically eliminates intratumoral *F. nucleatum*, thereby directly reversing *F. nucleatum*‐mediated immunosuppression while preserving gut microbiota homeostasis. Crucially, the killed *F. nucleatum* acts as an immunostimulant, promoting robust activation of antigen‐presenting cells (APCs) and enhancing infiltration of cytotoxic T lymphocytes (CTLs) within the tumor. This dual action—elimination of suppressive bacteria and immunogenic activation by bacterial remnants—effectively reprograms the immunosuppressive tumor microenvironment (TME) in colorectal cancer. This work proposes a therapeutic strategy for cancer that targets tumor‐associated bacteria to reprogram the TME, paving the way for more effective cancer treatments.

## Graphical abstract

Tumor‐related pathogenic bacteria can affect the treatment efficiency of tumors. Therefore, we have designed a nanoparticle that can accurately target tumor cells and related pathogenic bacteria, reverse the immunosuppressive effect of pathogenic bacteria on tumors, and synergistically enhance tumor treatment efficiency through sonodynamic therapy.

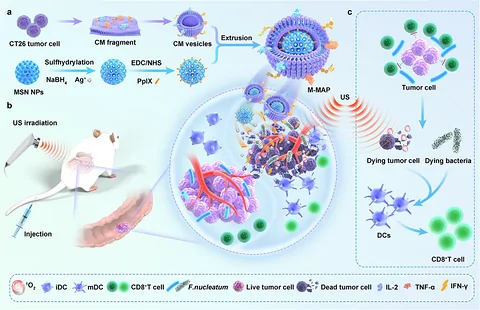

## 1 Introduction

The tumor microenvironment (TME), a dynamic and complex ecosystem surrounding tumors, critically influences tumor progression and resistance to therapy [1, 2, 3]. One of the most significant barriers to effective cancer treatment is the immunosuppressive TME, which limits the efficacy of current therapeutic strategies [4]. Emerging evidence highlights the role of microbiota in shaping the TME and influencing tumor progression [5]. While certain commensal bacteria enhance antitumor immunity, disruptions in the tumor‐associated microbiota [6, 7], characterized by the increased abundance of pathogenic species like *Fusobacterium nucleatum* (*F. nucleatum*), *Helicobacter hepaticus* (*H. hepaticus*), and *Peptostreptococcus anaerobius* (*P. anaerobius*), drive tumor progression by reshaping the TME and impairing immune responses [8, 9, 10, 11]. Among pathogenic bacteria, *F. nucleatum* is significantly connected to the advancement of CRC and resistance to therapies [12, 13, 14]. Mechanistically, *F. nucleatum* modulates the TME through multiple pathways, including TLR4‐MYD88 signaling and TIGIT‐mediated immune evasion, promoting chemoresistance and suppressing CD8^+^ T cell activity [12, 14, 15]. Its abundance correlates with poor clinical outcomes, highlighting its critical role in CRC pathogenesis. These findings underscore the importance of strategies to eliminate tumor‐associated pathogens for effective cancer treatments.

The current treatment of pathogenic bacteria mainly relies on various antibiotics [16]. However, systemic administration of antibiotics might lead to dysbiosis of the gut microbiota, which can induce unintended health complications [17, 18]. Precision approaches that target pathogenic bacteria within tumors are urgently needed. Nanotechnology offers a promising solution, enabling targeted pathogen elimination while preserving the microbiota balance, improving anti‐tumor immune response, and enhancing therapeutic efficacy [19, 20]. Recent advancements in nanoparticle‐based systems have demonstrated the potential to overcome therapy resistance by eliminating tumor‐colonizing bacteria [21, 22, 23]. For instance, dual‐cascade responsive nanoparticles amplified tumor treatment efficiency by eliminating bacteria within pancreatic cancer cells, thereby protecting chemotherapy drugs from bacterial metabolism [24]. *F. nucleatum*‐mimetic nanoparticles were designed by fusing bacterial membranes with colistin‐loaded liposomes. These nanoparticles deliver antibiotics specifically to tumor‐colonized bacteria, thereby re‐sensitizing *F. nucleatum*‐infected tumors to ICB therapy [25]. Our team has also researched the application of nanotechnology to eliminate specific pathogenic bacteria, *F. nucleatum*, and improve the efficiency of treatment for CRC [26, 27]. However, challenges remain in achieving specifically targeting and effectively reprogramming the TME to overcome immune suppression and activate robust antitumor immunity.

Herein, we employed mesoporous silicon nanoparticles (MSNs) as carriers, loaded with silver nanoparticles (Ag NPs) and sonosensitizer protoporphyrin (PpIX), and coated the CT26 tumor cell membranes. The resulting membrane‐camouflaged Ag@MSN‐PpIX NPs, called M‐MAP NPs, exhibited strong homologous targeting, enabling their preferential enrichment in colorectal tumors. Under US irradiation, the PpIX in M‐MAP NPs, which was the organic sonosensitizer as previous studies mentioned [28, 29], generated high levels of singlet oxygen (^1^O_2_), one kind of reactive oxygen species (ROS), leading to efficient tumor cell eradication. Simultaneously, with the enrichment of nanoparticles homologously targeted to tumor cells, the release of Ag NPs suppressed the growth of the tumor‐associated bacterium *F. nucleatum*, mitigating the pathogen‐induced immunosuppressive microenvironment. This intervention promoted the maturation of APCs (such as dendritic cells, or DCs), fostered the activation of CD8^+^ T cells in the tumor microenvironment, and ultimately enhanced antitumor immune responses. The strategy of specifically eliminating tumor pathogens, reversing the tumor immune microenvironment, and combining sonodynamic therapy (SDT) provides a possibility for the future treatment of CRC.

## 2 Results and Discussion

### 2.1 Synthesis of M‐MAP NPs With Sonodynamic Therapy Effect

**SCHEME 1:**
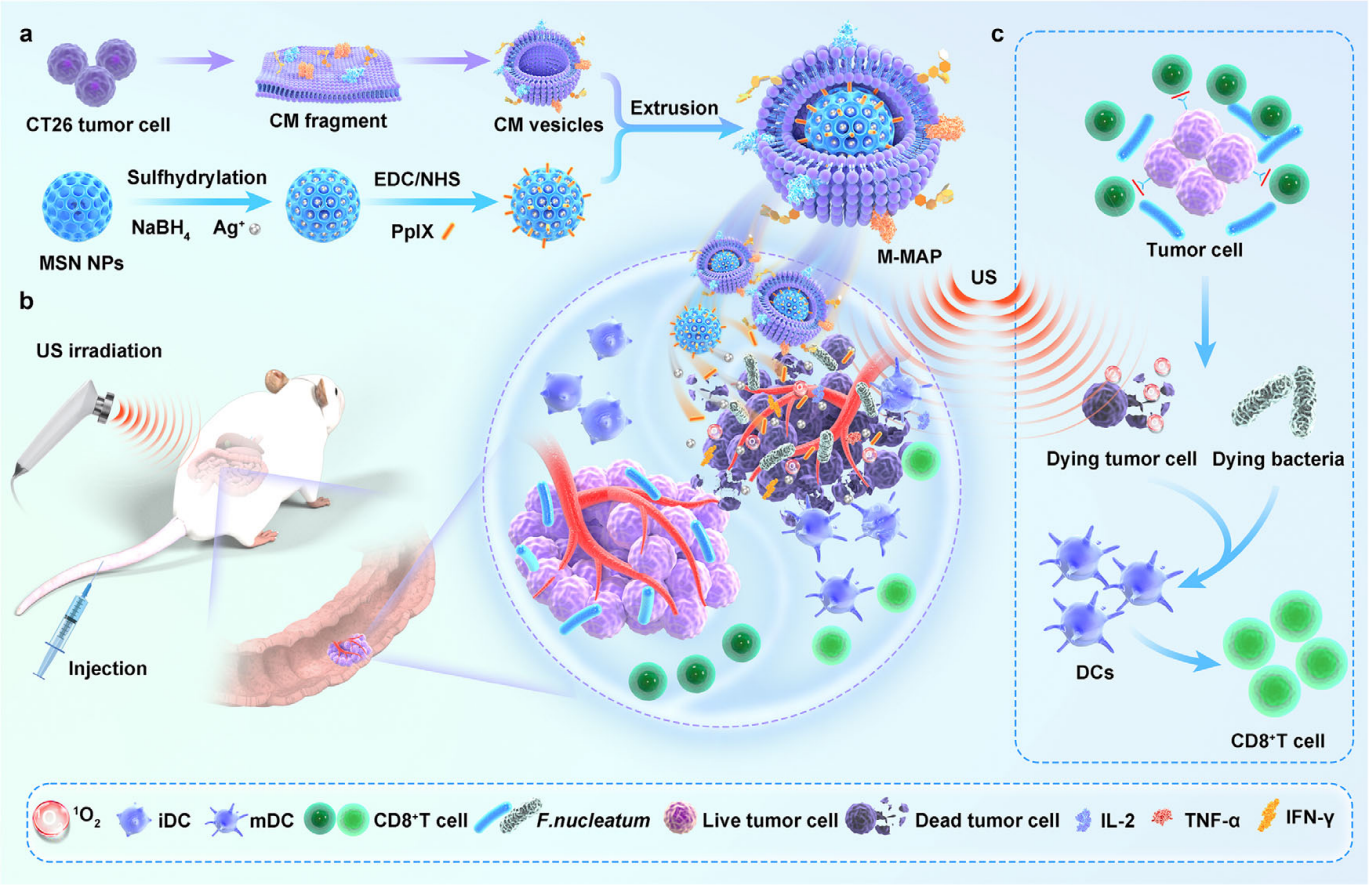
Outline of M‐MAP construction and its mechanism in *F. nucleatum*‐associated CRC treatment strategy. (a) Synthesis of MAP and M‐MAP. (b, c) Mechanistic cascade of M‐MAP+US treatment for CRC infected by *F. nucleatum*. The *F. nucleatum*‐infected TME establishes immunosuppressive conditions through bacterial‐induced antigen masking mechanisms. This microbial interference impedes DCs’ maturation by compromising antigen recognition capacity, subsequently hindering T cell infiltration into tumor tissues. Upon intravenous administration of M‐MAP nanoparticles followed by ultrasound (US) irradiation, the growth of *F. nucleatum* is inhibited. This promotes the maturation of DCs, facilitating antigen presentation. Simultaneously, CD8^+^ T cells are activated by IL‐2, leading to the release of TNF‐α and IFN‐γ, which synergistically enhance tumor treatment efficiency through SDT.

**FIGURE 1:**
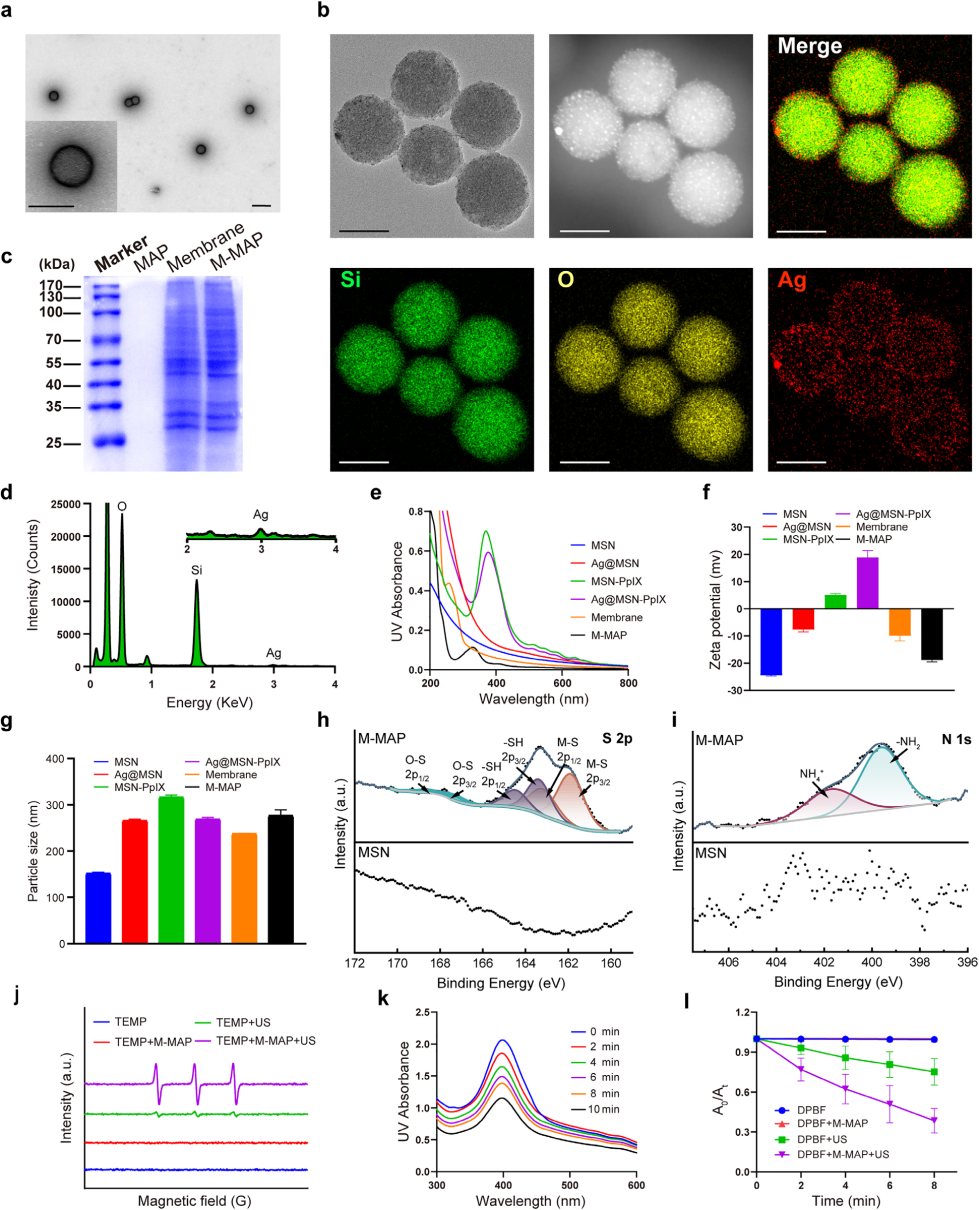
Evaluation of the structural, compositional, and physicochemical features of M‐MAP. (a) TEM images of M‐MAP (scale bar = 200 nm). The samples were negatively stained using uranyl acetate. (b) HAADF images and corresponding elemental mapping of M‐MAP (scale bar = 50 nm). (c) SDS‐PAGE protein analysis of Ag@MSN‐PpIX (MAP), CT26 cell membrane, and M‐Ag@MSN‐PpIX(M‐MAP). (d) The EDS spectra of M‐MAP. (e) UV‐vis absorption spectra of MSN, Ag@MSN, MSN‐PpIX, Ag@MSN‐PpIX, Membranae, and M‐Ag@MSN‐PpIX. (f) Zeta potential changes of MSN, Ag@MSN, MSN‐PpIX, Ag@MSN‐PpIX, Membranae, and M‐Ag@MSN‐PpIX (*n =* 3). (g) Hydrodynamic diameters of MSN, Ag@MSN, MSN‐PpIX, Ag@MSN‐PpIX, cancer cell membrane, and M‐Ag@MSN‐PpIX (M‐MAP). Data are presented as means ± SD (*n =* 3). (h‐i) XPS spectra of S 2p (h) and N 1s (i) in MSN and M‐MAP. (j‐l) The ability to generate singlet oxygen (^1^O_2_) in M‐MAP. (j) ESR spectra of M‐MAP with or without US. TEMP with US was used as a control comparison. (k) Time‐dependent DPBF absorption spectra of M‐MAP under US irradiation (1.0 MHz, 1.5 W cm^−2^, 50% duty cycle, every 2 min). (l) Time‐dependent DPBF degradation curves of M‐MAP with or without US (*n =* 3). Data are presented as means ± SD.

To specifically eliminate tumor pathogens and enhance tumor therapeutic effects, we designed a novel nanoplatform, M‐Ag@MSN‐PpIX (hereafter M‐MAP), composed of MSNs loaded with silver nanoparticles (Ag NPs) and protoporphyrin (PpIX), encapsulated in CT26 tumor cell membranes (Scheme 1). The synthesized MSN exhibited a uniform pore structure, which can operate as a storage facility for encapsulating guest cargos like antibacterial Ag NPs [30, 31]. To synthesize M‐MAP, Ag NPs were grown in situ within mesoporous MSNs by the sodium borohydride rapid reduction method, as we previously reported [32]. The sulfhydryl (‐SH) groups on the MSNs were functionalized to facilitate the coordination of silver ions (Ag^+^), enabling uniform incorporation of Ag NPs within the mesoporous matrix. Protoporphyrin (PpIX) was attached to the surface of the MSNs, followed by membrane coating using CT26 tumor cell membranes, facilitated by repeated extrusion through an Avanti mini‐extruder. The success of cell membrane encapsulation was validated through transmission electron microscopy (TEM) and sodium dodecyl sulfate‐polyacrylamide gel electrophoresis (SDS‐PAGE), as shown in Figures 1a–c. TEM imaging (Figure 1a) revealed that the M‐MAP nanoparticles exhibited a well‐dispersed and uniform size distribution, with a mean diameter of 78.1 ± 7.1 nm. The characteristic contrast associated with Ag NPs, due to their higher atomic number (*Z =* 47), was evident in high‐angle annular dark‐field (HAADF) imaging, confirming the successful loading of Ag NPs in the mesopores of the MSNs. Energy dispersive spectrometer (EDS) elemental mapping revealed the homogenous distribution of Si, O, and Ag within the nanoparticles (Figure 1b). Si, O, and Ag signals were also observed in the EDS spectrum of M‐MAP NPs (Figure 1d). In addition, ICP‐MS was used to detect the actual amount of Ag NPs loaded in M‐MAP NPs. We calculated that the encapsulation efficiency of Ag NPs was 69.1% and the loading capacity was 10.98% (w/w). Ultraviolet‐visible (UV‐vis) spectra confirmed the successful surface grafting of PpIX, with characteristic peaks observed in the spectra (Figure 1e). The encapsulation efficiency and loading capacity of PpIX were calculated to be 12.5% and 1.14 wt%, respectively. (Figure S1). The hydrodynamic size and zeta potential were conducted on M‐MDP nanoparticles to evaluate their stability and surface charge characteristics. The dynamic light scattering (DLS) results indicated a growth in the hydrodynamic diameter of M‐MAP and a zeta potential change from +18.88 mV to −18.83 mV, indicating successful encapsulation of negatively charged tumor cell membranes (Figure 1f,g). Moreover, the M‐MAP nanoparticles remained stable for up to one week after membrane encapsulation, as confirmed by DLS measurements (Figure S2). X‐ray photoelectron spectroscopy (XPS) was then used to verify the successful modification of thiol (‐SH) and amino (‐NH_2_) groups on MSNs (Figure 1h,i). which was critical for confirming Ag NPs in situ growth and PpIX conjugation. As shown in Figure 1h, there were no S 2p in the MSNs, but S 2p obviously appeared in M‐MAPs, which were modified with ‐SH (163.37 eV and 164.49 eV). And as shown in Figure 1i, the high‐resolution N 1s spectra of M‐MAPs revealed the presence of ‐NH_2_ (399.61 eV). Next, we assessed the capacity of M‐MAP to generate ROS, as in previous reports [28, 33]. The generation of singlet oxygen (^1^O_2_) was initially examined through electron spin resonance (ESR) assays under the intervention of ultrasound irradiation (1.0 MHz, 1.5 W cm^−2^, 50% duty cycle for 5 min). The ESR results demonstrated that M‐MAP generated a significantly higher amount of ^1^O_2_ compared to TEMP alone under ultrasound irradiation (Figure 1j). The quantitative analysis of ROS was then confirmed by the degradation of DPBF, a well‐established indicator of ^1^O_2_ activity. The reduction in DPBF absorbance at 410 nm following ultrasound treatment confirmed the formation of ^1^O_2_. After 8 min of ultrasound exposure, the absorbance of DPBF was decreased to 38.5% of its initial value (Figure 1k,l), suggesting efficient ROS production by M‐MAP.

### 2.2 Tumor‐Targeted M‐MAP NPs Enhance Sonodynamic Therapy Against Tumor Cells and Pathogenic Bacteria

**FIGURE 2:**
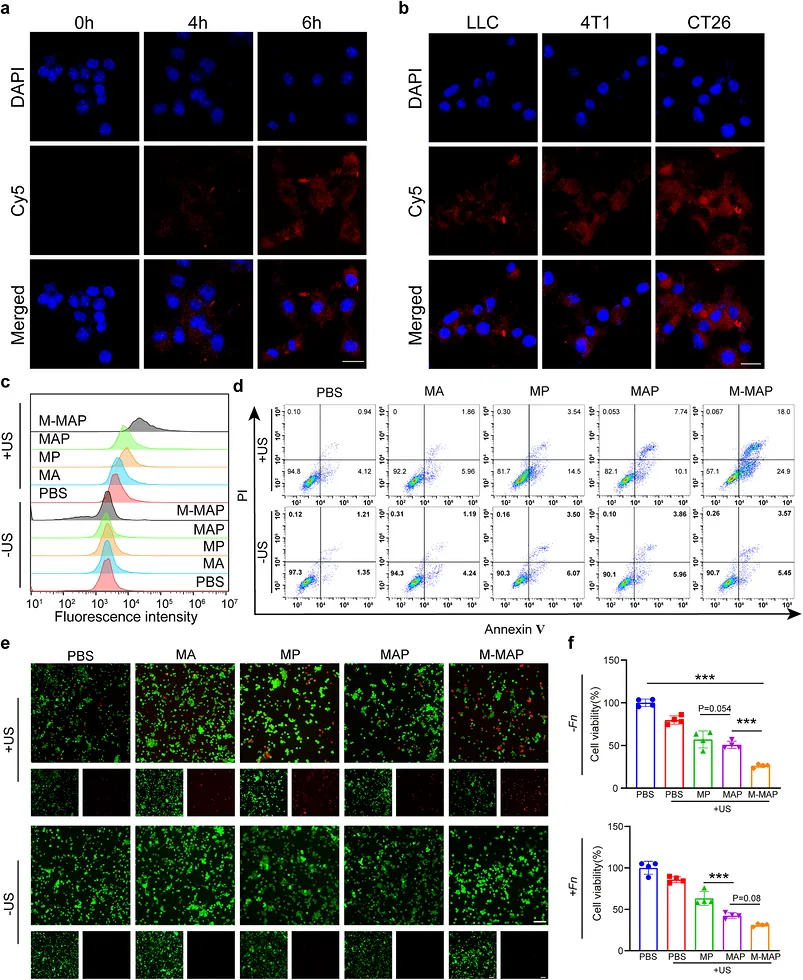
Tumor‐targeting M‐MAP NPs enhance in vitro SDT efficacy. (a, b) Representative LSCM images of cellular uptake at different times in CT26 (0, 4, 6 h) and different cell lines for 6 h (LLC, 4T1, and CT26); blue, nuclei stained with DAPI; red, M‐MAP labeled with Cy5 (scale bar = 20 µm). (c) In vitro cellular ROS fluorescence intensity of CT26 after different treatments with or without US. (d) Flow cytometry analysis for evaluation of apoptosis at various stages of different treatments. (e) Representative fluorescence micrographs of CT26 cells stained using Calcein‐AM/PI for live/dead cells (scale bar = 50 µm). (f) The cell viability of CT26 cells incubated without or with *F. nucleatum* (*Fn*) after different treatments (*n =* 4). The treatments are the same as the ROS detection experiment. Data are presented as means ± SD. *** *p* < 0.001.

Surface modification of MAP NPs with tumor cell membranes endows homologous targeting ability, as previously reported [34, 35, 36]. The cellular uptake of M‐MAP was evaluated by incubating Cy5‐labeled M‐MAP nanoparticles with CT26 cells. As shown in Figure 2a, the signal of Cy5 accumulated over time. The incubation of M‐MAP‐Cy5 with LLC cells (mouse Lewis lung carcinoma cells), 4T1 cells (mouse breast cancer cells), and CT26 cells (mouse colon tumor 26 cells) was conducted to assess if homotypic targeting contributed to its effective cell uptake. After 6 h, the cells were gathered for observation using laser scanning confocal microscopy (LSCM) and for analysis via flow cytometry. The results indicated that M‐MAP NPs were taken up more by homologous CT26 cells due to the homologous targeting effect (Figure 2b and Figure S3a). The quantitative results indicated that the fluorescence intensity in CT26 cells was 2.6 times that of LLC cells and 2.8 times that of 4T1 cells (Figure S3b). This result also supported the above conclusion that M‐MAP wrapped with CT26 tumor cell membranes could be taken up more by homologous cells.

SDT, serving as an exogenous stimulus, can lead to the production of quantities of ^1^O_2_. And previous studies have proved that redox imbalances in cancer cells that might result in tumor apoptosis [37]. To investigate the effect of SDT, 2′,′7′‐dichlorofluorescein diacetate (DCFH‐DA) was used to detect the generation of ^1^O_2_ in vitro. As illustrated in Figure S4, without PpIX or US irradiation, nanoparticles are unable to generate ROS. Additionally, significant ROS generation occurred in MP (MSNs loaded with Ag NPs)+US, MAP (MSNs loaded with Ag NPs and PpIX)+US, and M‐MAP+US groups, while the ROS levels in the other groups without US were lower (Figure 2c). Compared to the MAP+US group, the M‐MAP+US group demonstrated a more noticeable increase in ROS fluorescence signals, with the intensity rising by roughly 3.1‐fold (Figure S5). These findings confirmed that camouflaged cell membranes result in more M‐MAP being engulfed by tumor cells, thereby enhancing the SDT efficacy.

Because of the efficient generation of ROS by tumor cell membrane‐disguised M‐MAP, we determined whether M‐MAP would improve therapeutic efficacy. Initially, the cell viability of CT26 cells after being co‐incubated with MSNs, MA NPs, MP NPs, MAP NPs, and M‐MAP NPs at concentrations ranging from 0 to 20 µg/mL could remain above 80.0%, indicating the negligible cytotoxicity of these nanocomposites (Figure S6). As showed in Figure S7, the SDT effect is dependent on PpIX concentration (Figure S7a), ultrasound intensity (Figure S7b), and irradiation time (Figure S7c). Based on these findings and prior studies, the parameters for subsequent experiments were set at 1.0 MHz and 1.5 W cm^−2^. This selection aims to balance preclinical proof‐of‐concept with potential clinical applicability. We acknowledge that treatment of deeply located or acoustically complex tumors may require further image‐guided parameter personalization. The parameters used here serve as a foundational benchmark; future clinical protocols could adapt the frequency, intensity, and exposure time based on individual patient and tumor characteristics. And the practical application of ultrasound‐mediated therapy for orthotopic colon tumors must consider potential energy attenuation through the abdominal wall. This challenge can be mitigated through careful experimental design, including the use of lower‐frequency ultrasound for deeper tissue penetration and optimized focused‐beam protocols for precise energy delivery. Such strategies are crucial to ensure the effective activation of sonosensitizers at the target site while preserving surrounding healthy tissues. Subsequently, Annexin V‐FITC and propidium iodide (PI) staining were used to assess apoptosis at various stages (Figure 2d). The data revealed that M‐MAP with SDT triggered significantly higher levels in both early and late apoptosis (42.9%) compared to MAP (17.84%). Subsequently, the findings were corroborated by fluorescence images of CT26 cells, which were co‐stained with calcein acetoxymethyl ester (calcein‐AM) and PI to differentiate between live (green) and dead (red) cells visually, and the results indicated the excellent increased SDT efficiency of M‐MAP under US (Figure 2e).

Because the therapeutic effect of CRC can be influenced by the presence of *F. nucleatum*, we assessed whether silver nanoparticles in M‐MAP could synergistically interact with SDT to inhibit the growth of *F. nucleatum* and enhance the effectiveness of tumor therapy. The *F. nucleatum*‐infected CT26 tumor cell model (MOI = 100:1) was established to investigate the therapeutic efficacy of M‐MAP in vitro. Then, cytotoxicity assessment was performed with the CCK‐8 assay, revealing that the MAP+US group significantly suppressed the proliferation of *Fn*/CT26 cells to 42.41%, compared to 63.17% in the MP + US group (Figure 2f), indicating that Ag nanoparticles could overcome the proliferative effect of *F. nucleatum* on the tumor. Moreover, after treatment with M‐MAP+US, cell viability dropped markedly to 31.09%, outperforming the MAP+US group, which showed the viability of 42.41% (Figure 2f). Taken together, tumor‐targeted M‐MAP NPs with SDT overcame the ability to promote tumor proliferation by *F. nucleatum* and heightened the killing ability of *F. nucleatum*‐infected CT26 tumor model.

### 2.3 In Vitro Antibacterial Effect

Subsequently, the killing ability of M‐MAP NPs against bacteria alone was tested. As previous studies illustrated, the sustained release of silver ions was the critical factor in killing microbes. The accumulated Ag^+^ could cause cell membrane denaturation and even penetrate the bacterial cell wall to induce bacterial death [38, 39]. Therefore, we examined the ability of M‐MAP NPs to continuously release Ag^+^ under different US intensity irradiations by using ICP‐MS. The results showed that M‐MAP NPs could sustain release Ag^+^ under US irradiation and were US‐intensity dependent (Figure S8a). This is likely due to US‐generated ROS, which promotes the oxidation of Ag nanoparticles and consequently accelerates Ag^+^ release. In addition, different pH levels were set to detect the release profile of MAP NPs in extended time (0, 12, 24, 48, 72, 96 h). As shown in Figure S8b, under neutral conditions (pH = 7.4), Ag^+^ released slowly. When pH changed to acidic conditions close to TME, the release of Ag^+^ increased. Different interventions were used to treat *F. nucleatum* in its logarithmic growth phase overnight for a more intuitive evaluation. After the US, bacteria were harvested and spread on Columbia blood agar plates. Compared to the PBS group, the groups containing Ag NPs exhibited a notable reduction in colony count (Figure S9a). At the same time, the account of bacterial colonies and OD600 nm absorbance of bacteria showed that the inhibition caused by Ag NPs alone was more apparent than that caused by SDT (Figure S9b and c). Therefore, the bacterial viability was detected with different concentrations of Ag NPs at various times. We found that the antibacterial effect of Ag NPs was concentration‐ and time‐ dependent (Figure S9d). The bacterial live/dead staining experiment yielded results consistent with those observed in the plate experiment, where *F. nucleatum* was destructively killed by M‐MAP+US treatment, as evidenced by the red staining in the CLSM images (Figure S10). These results proved that M‐MAP with US could suppress the growth of *F. nucleatum* effectively. In conclusion, the continuous release of silver ions under US irradiation could inhibit the bacterial growth.

### 2.4 Therapeutic Efficacy of M‐MAP NPs Under US in *F. nucleatum*‐Infected CRC models

The biocompatibility of nanomaterials is vital for their potential use in clinical settings. Therefore, the biological safety of MP, MAP, and M‐MAP was assessed by mice body weight, hematological biochemistry, and histological assessment of mice after receiving intravenous injection of saline, MP NPs, MAP NPs, and M‐MAP NPs (the PpIX dose of 10 mg/kg, 100 µL) for 14 days. No significant body weight loss was observed compared with the control group (Figure S11). Biochemical indicators, including aminotransferase (AST), alanine aminotransferase (ALT), alkaline phosphatase (ALP), blood urea nitrogen (BUN), and creatinine (CRE), did not exhibit any significant pathological alterations (Figure S12). In addition, the toxicity of nanomaterials administered systemically was examined by conducting hematoxylin and eosin (H&E) staining on primary organs, including heart, liver, spleen, lungs, and kidneys. There was no apparent pathological toxicity in the histological analysis (Figure S13). All the results proved high biocompatibility and biosafety of the MP, MAP, and M‐MAP for further treatment in vivo.

Then, to detect the drug distribution, the fluorescent molecule Indocyanine Green (ICG) was labeled on M‐MAP to construct ICG‐M‐MAP. After intravenous injection of ICG‐M‐MAP for 0, 12, 24, 48, and 72 h, CT26 tumor‐bearing mice were sacrificed for ex vivo fluorescence imaging (Figure S14a). It was depicted that the fluorescence in tumor tissues gradually strengthened as time went by, reaching the strongest at 48 h and beginning to weaken. Additionally, M‐MAP may be metabolized through the liver, as fluorescent signals also appear in the liver. Besides, the homologous targeting function of M‐MAP was investigated in mice bearing CT26, 4T1, and LLC tumors. As depicted in Figure S14b and c, after 24 h of intravenous injection, the tumor tissues in CT26 mice exhibited the highest fluorescence signal when compared to 4T1 and LLC mice. Furthermore, the sustained accumulation of Ag NPs in tumor tissue was the key to inhibiting *F. nucleatum*. We hence measure the contents of Ag NPs in dissected tumors at different time points after tail vein injection of M‐MAP NPs (0, 12, 24, 48, 72 h) by ICP‐MS. As shown in Figure S15, the content of Ag NPs in the tumor site gradually increased and persisted with prolonged time durations. The outcome was consistent with the in vitro experiment mentioned above, demonstrating the tumor cell targeting potential of M‐MAP.

Inspired by the effective therapy in vitro and high compatibility in vivo, the tumor treatment potential of M‐MAP was immediately explored in vivo. The *F. nucleatum*‐infected CT26 subcutaneous tumor model was established as described in previous reports [40, 41]. Fluorescence in situ hybridization (FISH) technology was used to measure the colonization of *F. nucleatum* to make sure of the successful establishment of the animal mode (Figure S16). Then, CT26/*Fn* subcutaneous tumor‐bearing mice were randomly assigned into nine groups, including control (without *F. nucleatum* infection), *Fn* (without treatment), US, MP, MP+US, MAP, MAP+US, M‐MAP, and M‐MAP+US. The formulations were subcutaneous injections into mice (Figure S17a). As shown in Figure S17b, tumors infected with *F. nucleatum* exhibited a larger tumor volume than those in the control group, implying that *F. nucleatum* could contribute to the development of CRC. Compared to the MP+US treatment group, the MAP+US group led to a decrease in tumor sizes, implying that Ag NPs in MAP could counter the tumor‐promoting effect of *F. nucleatum* (Figure S17c). The tumor volumes of the M‐MAP +US group were smaller than those of the MAP +US group, proving the strong and effective antitumor effects achieved by the combination of Ag NPs, homologous targeting ability, and SDT. Following the sacrifice of the mice, the tumors were extracted for H&E staining, terminal deoxynucleotidyl transferase‐mediated fluorescein‐dUTP nick‐end labeling staining (TUNEL), and immunohistochemical staining of Ki‐67, proving that M‐MAP enhanced the efficacy of tumor treatment by clearing *F. nucleatum* (Figure S18).

**FIGURE 3:**
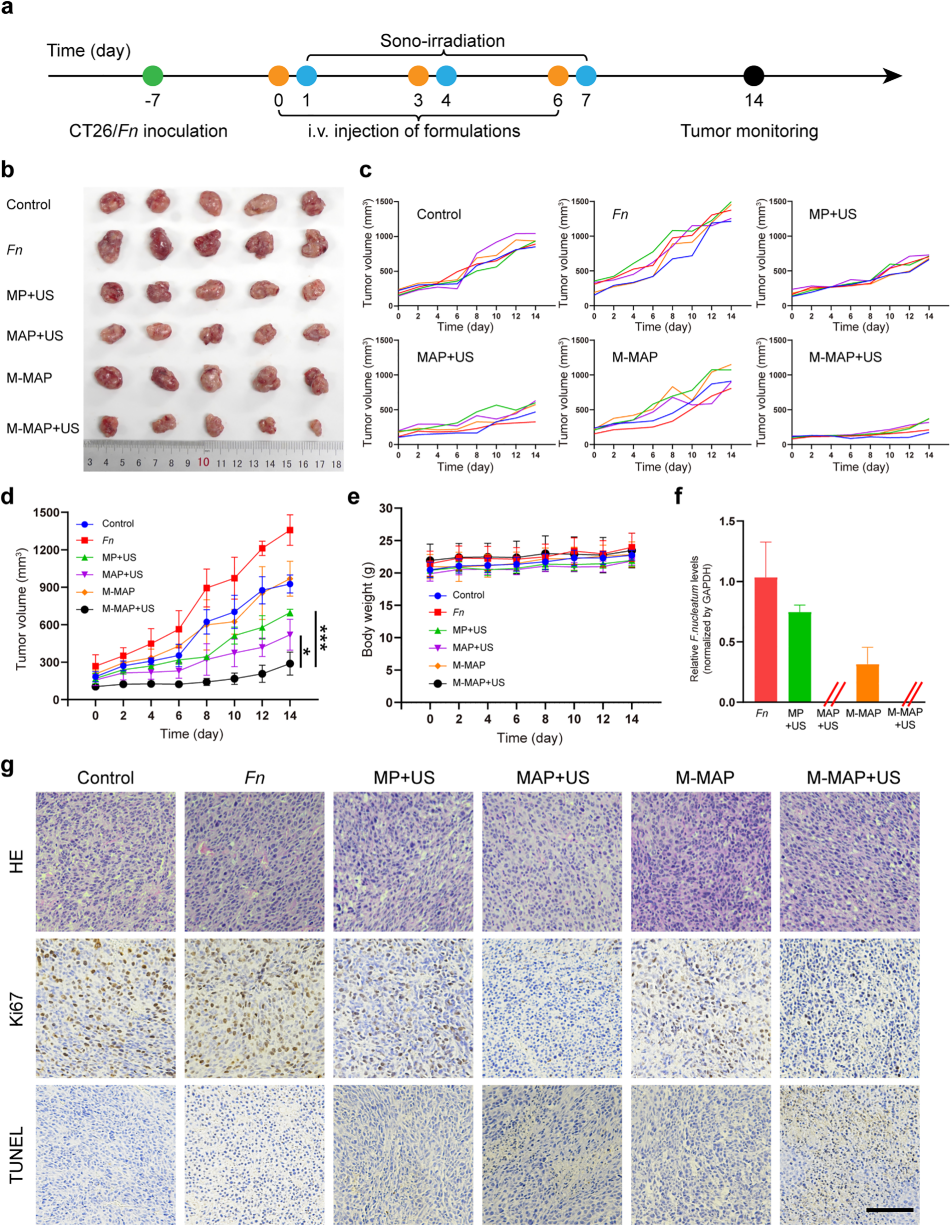
Intravenous administration of M‐MAP can significantly hinder the growth of the tumor in a CT26/*Fn* CRC subcutaneous model. (a) Diagrammatic representation of mice with tumors treated with various formulations under US exposure. (b) Photograph images of excised tumor sections from various groups on the 15th day. (c, d) Tumor‐volume evolutions of different groups during the treatment period. (e) Curves of body weight of different treatments during the therapeutic time. (f) The relative *F. nucleatum* abundance in tumor tissues after treatments: data was normalized by GAPDH (red slash represents undetected). (g) Representative photographs of H&E, Ki67, and TUNEL staining of excised tumor sections from different treatment groups (bar = 100 µm). Values are presented as means ± SD (*n =* 5). *p < 0.05, *** *p* < 0.001.

After that, the therapeutic efficacy of intravenous injection of M‐MAP was validated. As previously reported and based on our experimental results, US irradiation was necessary for SDT. Therefore, when setting the grouping, we treated the US as a constant and randomly allocated CT26/*Fn* subcutaneous tumor‐bearing mice into six groups: control, *Fn*, MP+US, MAP+US, M‐MAP, and M‐MAP+US. As illustrated in Figure 3a, the administration of MP, MAP, and M‐MAP was done i.v. at a dose of 10 mg/kg of PpIX on days 0, 3, and 6. After intravenous injection of nanoparticles for 24 h, the mice were subjected to US for 10 min. The tumor volumes and the mice's body weight were monitored every 2 days. As shown in Figure 3b‐d, M‐MAP+US could effectively suppress the growth of the CT26/*Fn* tumor model. In detail, the tumor inhibition ratio in the M‐MAP+US group was higher than the MAP+US group (31.2% vs. 56.1%), which indicated that the homologous targeting ability of tumor cell membranes enabled a greater number of nanoparticles to enter tumor cells, boosting therapeutic effects. No significant alterations were observed in the mice's body weight (Figure 3e). Following the various treatments, the mice were euthanized on day 14, and tumors were harvested for further study. Photographs of the tumors were taken (Figure 3b), showing results similar to subcutaneous injection treatment. Using quantitative real‐time PCR, the levels of *F. nucleatum* in tumor tissues after treatments were evaluated. Compared with the MP+US group, the relative abundance of *F. nucleatum* decreased significantly in MAP+US or M‐MAP+US, revealing a marked inhibition of *F. nucleatum* in tumor sites due to Ag NPs (Figure 3f, Table S1). We speculated that the reason for this phenomenon might be the efficient killing effect of Ag NPs combined with SDT on bacteria. While the observed reduction in *F. nucleatum* load highlights the potent short‐term antibacterial effect of Ag NPs combined with SDT, this study did not assess the potential for bacterial relapse or the development of resistance. Future work should include longitudinal monitoring of bacterial load over extended post‐treatment periods to evaluate recurrence, alongside in vitro studies to examine any adaptive responses of the bacteria to treatment. Moreover, H&E, TUNEL, and Ki‐67 immunohistochemical staining results exhibited that the M‐MAP+US treatment group had the most pronounced cell damage and apoptosis, coupled with the minimal proliferation of tumor cells (Figure 3g).

### 2.5 M‐MAP NPs Under the US Remodeled the Tumor Immune Microenvironment

**FIGURE 4:**
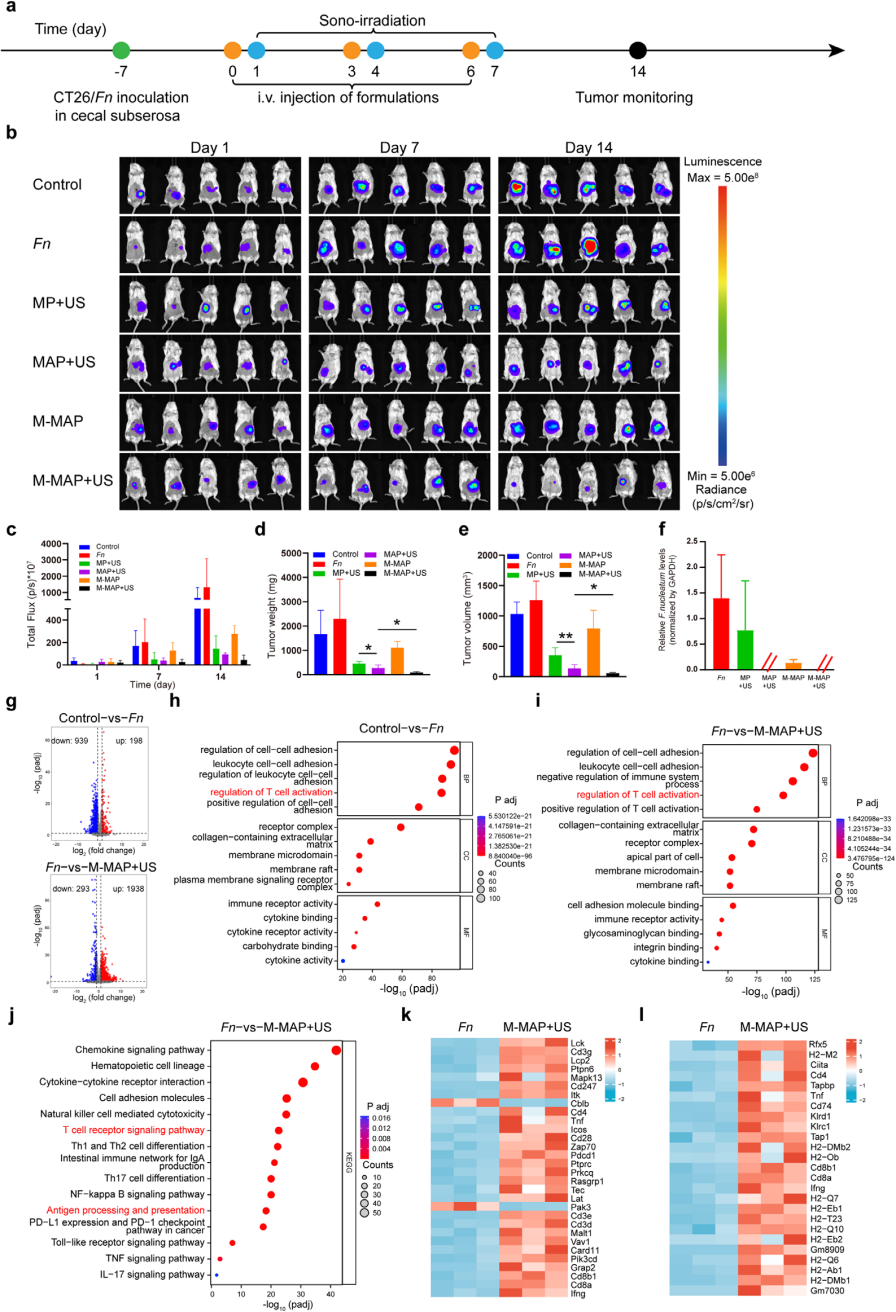
M‐MAP under US can effectively delay the progression of the in situ CT26‐luc/*Fn* tumor model of CRC by systemic immune response activation. (a) Timeline of a cecum orthotopic implantation model establishment and tumor‐bearing mice using various formulations subjected to US. (b) In vivo bioluminescent imaging of the tumor‐bearing mice of different groups was obtained using IVIS Spectrum at days 1, 7, and 14 after model establishment. (c) Quantification of the tumor bioluminescence signal of various treatments. (d) Tumor weight and (e) volume measured after treatment. (f) The relative *F. nucleatum* levels in the tumor after treatments: data were normalized by GAPDH (red slash represents undetected). (g) A volcano plot illustrated the overall distribution of regulated genes, with the x‐axis showing fold change in gene expression across different samples and the y‐axis indicating statistical significance (padj) of gene expression differences. Red, blue, and grey dots indicate genes that are upregulated, downregulated, and show no significant change, respectively. (h, i) GO analysis of the DEGs in (h) control versus *Fn* and (i) *Fn* versus M‐MAP+US (Biological Process, BP; Cellular Components, CC; Molecular Function, MF). (j) The enrichment scatter plot illustrated KEGG enrichment analysis for selected immune‐related KEGG terms from *Fn* versus M‐MAP+US. (k,l) Heat map showing gene expression status from *Fn* versus M‐MAP+US in (k) T cell receptor signaling pathway and (l) Antigen processing and presentation KEGG pathway. Values are presented as means ± SD (*n =* 5). *p < 0.05, **p < 0.01.

Furthermore, to better simulate the TME of CRC, a mouse cecal in situ colorectal tumor model infected with *F. nucleatum* was established. After 7 days of establishment, random assignment was used to divide the mice into six groups: control, *Fn*, MP+US, MAP+US, M‐MAP, and M‐MAP+US. Then, tumor progression was observed through bioluminescence imaging (Figure 4a). The fluorescence signal was found to be stronger in the *F. nucleatum*‐infected group than in the control group, indicating that *F. nucleatum* possesses the ability to enhance colorectal tumor proliferation (Figure 4b). Meanwhile, MAP+US treatment substantially inhibited the tumor growth, which was stronger than the MP+US group. Furthermore, M‐MAP+US treatment markedly reduced the spread of tumor invasion (Figure 4b). Quantitative analysis results of the tumor bioluminescence signals of various treatments could more objectively demonstrate the tumor suppressive ability of M‐MAP+US (Figure 4c). The mice were euthanized on day 14, and tumors were harvested for further examination. The tumor volume (Figure 4d) and tumor weight (Figure 4e) were recorded and statistically analyzed. The findings aligned with the statistics of the fluorescence signal. The relative *F. nucleatum* levels in tumors following various treatments were measured using quantitative Real‐time PCR. MAP+US and M‐MAP+US groups did not detect the presence of *F. nucleatum* (Figure 4f, Table S1). The reason we speculated might be that *F. nucleatum* was not the dominant strain in mice, and consistently existing high concentrations of silver ions had a good killing effect on it. Moreover, the curative effect was proven by H&E, TUNEL, and Ki‐67 staining, demonstrating that the M‐MAP+US group enhanced tumor treatment efficiency by inhibiting *F. nucleatum* (Figure S19). To systematically characterize the antitumor mechanisms of M‐MAP under US on the *Fn*‐infected CT26‐luc in situ cecum tumor model, tumor tissues from control, *Fn*, and M‐MAP+US groups were collected for transcriptome sequencing analysis. The analysis revealed that 939 genes were expressed at lower levels and 198 genes at higher levels in the *Fn* group compared to the control. While there were 293 genes downregulated and 1938 genes upregulated between *Fn* and M‐MAP+US groups (Figure 4g). An analysis of Gene Ontology (GO) enrichment and Kyoto Encyclopedia of Genes and Genomes (KEGG) pathways for regulated genes was performed. As shown in Figure 4h, compared to the control, the *Fn* group was significantly enriched in genes associated with the regulation of T cell activation. Compared with *Fn*, the M‐MAP+US group was prominently enriched in genes related to T cell activation (Figure 4i). Interestingly, these results indicated that the underlying antitumor mechanisms of M‐MAP under US might be associated with immune response. Furthermore, from the GO enrichment analysis results, the most significant top 5 terms in control vs. *Fn* and *Fn* vs. M‐MAP+US were also related to immune response (Figure S20a, b). To better reveal the potential antitumor mechanism of M‐MAP+US, immune‐related KEGG terms were analyzed. As shown in Figure 4j, compared with *Fn*, differentially expressed genes (DEGs) of the M‐MAP+US group were enriched in immune signaling pathways, innate immune response, and adaptive immune response, such as antigen processing and presentation and T cell receptor signaling pathways. Then, we selected T cell receptor signaling pathway and antigen processing and presentation‐related genes, and expression levels of DEGs were displayed in heatmaps (Figure 4k,l). Besides, as shown in Figure S21a and b, *F. nucleatum* could down‐regulate antigen processing and presentation‐related genes, while M‐MAP+US could up‐regulate antigen processing and presentation‐related genes. Gene set enrichment analysis (GSEA) was performed on DEGs based on GO enrichment and the KEGG pathway analysis. The results were consistent with the former, showing that *F. nucleatum* might inhibit immune response while M‐MAP+US could reverse the suppression induced by *F. nucleatum* (Figure S22–27). The protein‐protein interaction networks were used to analyze the associated functional genes (Figure S28). These proteins played a role in managing the immune response.

**FIGURE 5:**
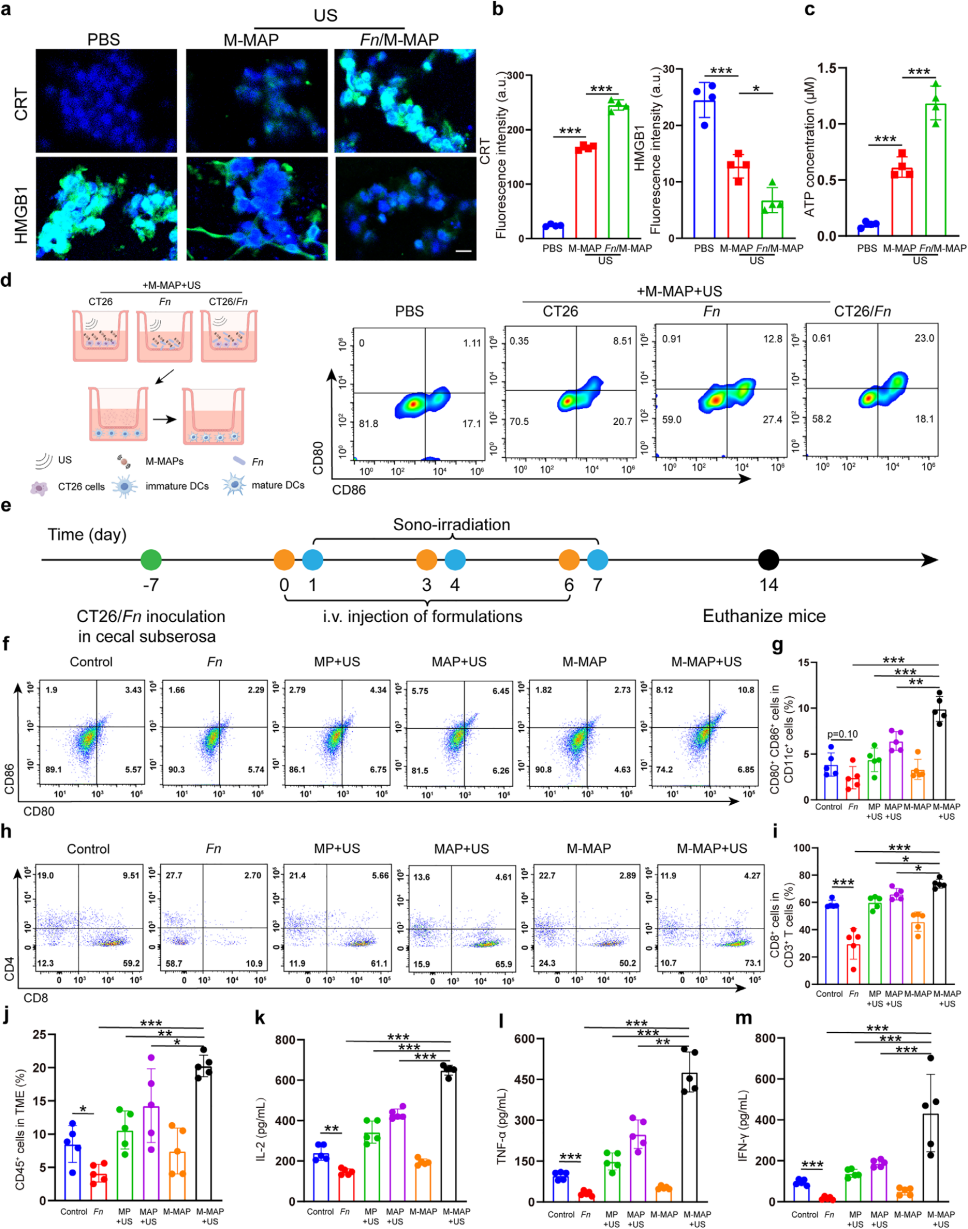
M‐MAP NPs under the US remodeled the tumor immune microenvironment. (a) Representative fluorescent images of CRT and HMGB1 in CT26 incubated with different conditions. (b) Fluorescence intensity of CRT and HMGB1 in CT26 under different conditions. (c) The extracellular ATP concentration of CT26 cells with different experimental conditions (*n =* 4). (d) Schematic representation of the experimental setup for in vitro BMDC maturation, utilizing a transwell system with CT26 cancer cells and *F. nucleatum* in the upper chamber and BMDCs in the lower chamber. US conditions were consistent with the conditions of the cell experiments. (By Figdraw). Representative flow cytometric plots of CD80^+^CD86^+^ cells in BMDC cells of various treatments. (e) Timeline of a cecum orthotopic implantation model establishment and various treatments under US. (f) Representative flow cytometric plots and (g) quantitative analysis of CD80^+^CD86^+^ cells in CD11c^+^ cells of the spleen after various treatments (*n =* 5). (h) Representative flow cytometric plots and (i) quantitative analysis of CD8^+^ T cells in CD3^+^ T cells in tumor sections from different groups (*n =* 5). (j) Quantitative analysis of the amount of CD45^+^ cells in the TME of different groups. (k) Cytokine levels of IL‐2, (l) TNF‐α, and (m) IFN‐γ in peripheral blood were measured through ELISA between different treated groups (*n =* 5). Data are presented as means ± SD. *p < 0.05, **p < 0.01, and ***p < 0.001.

According to transcriptome sequencing data, activating APCs is essential for initiating immune responses. To elucidate the immunogenic mechanisms underlying dendritic cell activation, we investigated the release of key damage‐associated molecular patterns (DAMPs) and subsequent antigen presentation. As shown in Figure 5a,b, treatment with M‐MAP + US in the presence of *F. nucleatum* significantly induced the extracellular release of high‐mobility group box 1 (HMGB1) from dying CT26 cells and enhanced the surface exposure of calreticulin (CRT). Consistent with these findings, adenosine triphosphate (ATP) levels in the cell culture supernatant were also markedly elevated (Figure 5c). We further evaluated the functional impact of DAMP release and bacterial killing on dendritic cell maturation using a transwell co‐culture system (Figure 5d). M‐MAP NPs were incubated separately with CT26 cells, *F. nucleatum*, or CT26 cells pre‐infected with *F. nucleatum* (MOI = 100:1) in the upper chamber for 6 h, followed by ultrasound irradiation. After removal of the upper chamber, bone marrow‐derived dendritic cells (BMDCs) in the lower chamber were cultured overnight and analyzed by flow cytometry. The proportion of matured dendritic cells (CD11c^+^CD80^+^CD86^+^) in the *F. nucleatum*/CT26 co‐culture group reached 24.93%, representing a 3.6‐fold and 1.8‐fold increase compared to the CT26‐only and *F. nucleatum*‐only groups, respectively (Figure S29 and Figure S33a). These results indicate that M‐MAP‐mediated sonodynamic therapy not only induces immunogenic cell death accompanied by DAMP release but also enhances antigen exposure through bacterial elimination, thereby synergistically promoting dendritic cell maturation and antigen presentation. Then, as shown in the timeline in Figure 5e, immune cells in vivo were evaluated by collecting tumor tissues and spleens from mice of various treatments (control, *Fn*, MP+US, MAP+US, M‐MAP, and M‐MAP+US) for flow cytometry measurement. In comparison to the control group, the spleens of the *Fn*‐infected group exhibited a reduced percentage of mature DCs (percentage of CD80^+^CD86^+^DCs in CD11c^+^ cells) (Figure 5f,g, and Figure S33b), indicating that *F. nucleatum* influenced antigen presentation and led to immune suppression. Compared to other treatments, mature DCs were most prevalent in the M‐MAP+US group (percentage of CD80^+^CD86^+^DCs in CD11c^+^ cells) in mice spleen, suggesting that M‐MAP+US could reverse the immune suppression by *F. nucleatum*. As is well known, T cell infiltration in tumors is essential for adaptive antitumor immune response. In our experiments, *F. nucleatum* influenced the tumor immune microenvironment and reduced the infiltration of immune cells (CD45^+^ cells and CTLs) at the tumor sites (Figure 5h–j). While M‐MAP+US could reverse the immune inhibition and activate the immune response. Meanwhile, the M‐MAP+US group showed significantly higher T cell infiltration in TME, especially CD8^+^ T cells (Figure 5h,I, and Figure S33c). Interleukin‐2 (IL‐2) plays a key regulatory role in the function of CD8^+^ cytotoxic T lymphocytes by promoting expansion and cytotoxic capability [42, 43]. Therefore, the serum cytokine levels of IL‐2 in different treatments were measured by enzyme‐linked immunosorbent assay (ELISA). The results showed that the level of IL‐2 was up‐regulated after treatment by M‐MAP with US (Figure 5k), implying that M‐MAP+US would produce more IL‐2, which can promote activation of CTLs. Activated CTLs can secrete cytokines with cytotoxic ability, such as interferon‐γ (IFN‐γ) and tumor necrosis factor‐α (TNF‐α) [44, 45]. Subsequently, we determined the cytokine levels of IFN‐γ and TNF‐α from various groups of mice by using ELISA. As shown in Figure 5l,m, the serum cytokine levels of TNF‐α and IFN‐γ of the M‐MAP+US group were the highest compared with other groups, suggesting that M‐MAP+US can promote the activation of CTLs and secrete an accumulating number of cytotoxic cytokines to combat the tumor. Collectively, M‐MAP NPs with the synergistic effect of SDT can reverse the tumor immunosuppressive microenvironment induced by *F. nucleatum*, promote antigen processing and presentation, stimulate the activation of CTLs infiltrating in the TME, and optimize the efficiency of cancer treatment.

### 2.6 M‐MAP NPs Under US Have Long‐Term Effectiveness of Tumor Therapy

**FIGURE 6:**
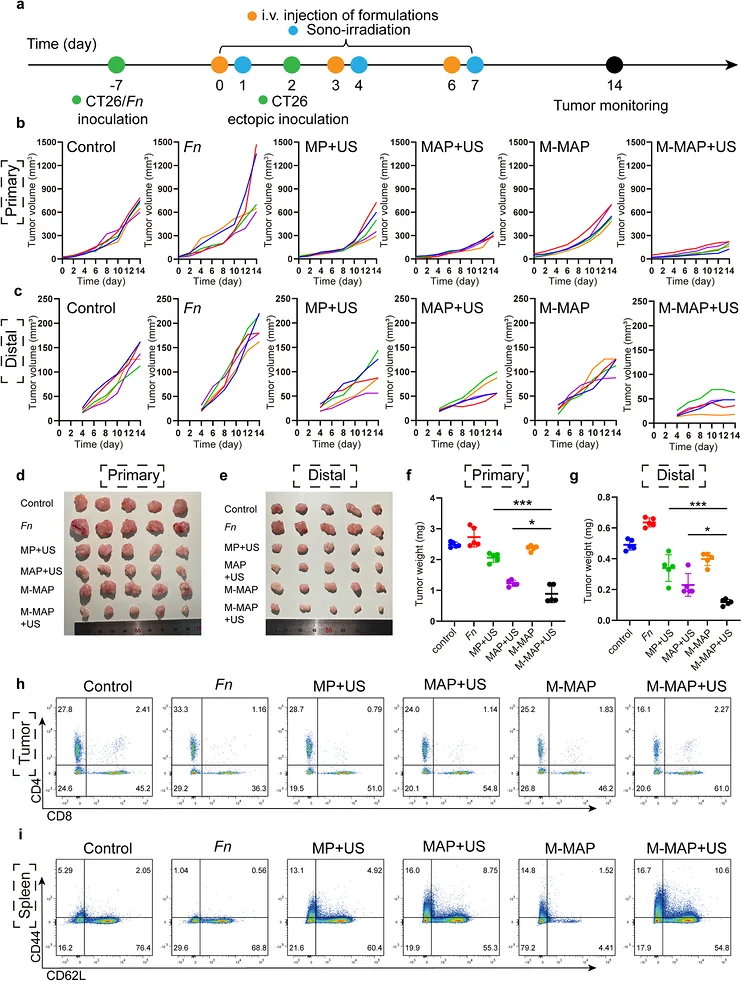
M‐MAP NPs under the US have long‐term effectiveness of the activated immune response. (a) The experimental schedule of M‐MAP NPs under US for long‐term therapy effectiveness on the bilateral CT26 tumor model. individual tumor volume curves of primary (b) and distal (c) tumors after various treatments. Photographs of dissected primary (d) and distal (e) tumors after different treatments. Tumor weight of primary (f) and distal (g) dissected tumors on the last day. (h) The representative flow cytometric plots of CD4^+^/CD8^+^ T cells in distal tumors from various experimental groups. (i) The representative flow cytometric plots of memory T cells in spleens from different experimental groups. Data are presented as means ± SD (*n =* 5). *p < 0.05, **p < 0.01, and ***p < 0.001.

Long‐term immunologic memory is a vital factor in elevating tumor immunotherapy [46]. Hence, we constructed a bilateral CT26 tumor model as depicted in Figure 6a. As shown in Figure 6b,d and f, M‐MAP+US could significantly suppress the primary tumor growth compared with other groups. And images of H&E staining and immunohistochemistry (IHC) staining of Ki‐67 showed that primary tumor tissue can be disrupted by M‐MAP+US (Figure S30). Furthermore, our previous work found that the colonization of *F. nucleatum* in the tumor activates the TLR4/MYD88 pathway. Therefore, we measured the expression of TLR4 and MYD88 in primary tumor sites by IHC staining. As shown in Figure S30, the M‐MAP+US group would decrease the expression of TLR4 and MYD88. We concluded that M‐MAP NPs under US irradiation inhibited the TLR4/MYD88 pathway induced by *F. nucleatum* in CRC cells, thereby suppressing CRC cells’ proliferation. Benefiting from the activated immune response, the growth of distal tumors in the M‐MAP+US group was inhibited the greatest among all groups (Figure 6c,e and g). For the sake of proving the anti‐tumor immune response ability of M‐MAP+US, immune cells in tumors and spleens were examined. For the sake of proving the anti‐tumor immune response ability of M‐MAP+US, immune cells in tumors and spleens were examined. First, we detected the regulatory T cells (Tregs) in primary tumor tissue, which inhibit anti‐tumor immune response and promote immune escape. We found that the colonization of *F. nucleatum* in tumors induced Treg infiltration to construct an immunosuppressive tumor environment. And M‐MAP NPs under US irradiation decreased the number of Tregs, which might help the anti‐tumor immune response (Figure S31 and Figure S33e). Then, we detected the immune cells in distal tumor tissue. As shown in Figure 6h and S32a, the infiltration of CD8^+^ T cells improved the greatest in the M‐MAP+US group compared with other groups. Meanwhile, central memory T (CD62L^+^, CD44^+^) cells were most pronounced in spleens of M‐MAP+US group, which played a key role in the long‐term effectiveness of anti‐tumor immune therapy (Figure 6i, Figure S32b, and Figure S33d). Owing to the long‐term activated immune response of M‐MAP under the US combined with antibacterial ability, the effectiveness of tumor therapy improved significantly.

### 2.7 Systemic Administration of M‐MAP NPs Maintained Intestinal Flora Balance in Mice

Ecological disturbances could substantially affect the abundance of bacterial communities in the gut, leading to a significant reduction in taxonomic richness, diversity, and evenness, which may facilitate the occurrence and progression of various diseases, including cancer [47, 48]. Thus, we further investigated whether systemic administration of M‐MAP NPs has an adverse effect on intestinal flora balance in mice. The *Fn*‐infected CT26‐luc in situ cecum tumor model was used, and the fecal samples from different treatments were collected. The overall structure of the gut microbiota was profiled using 16S rRNA gene sequencing. According to alpha diversity analysis, there was no notable difference in microbiota community richness (observed operational taxonomic units, OTUs) (Figure S34a) and diversity (Chao and Shannon indices) (Figure S34b, c) between the control and M‐MAP+US groups. The analysis of beta diversity using principal coordinates analysis (PCoA) depicted that the M‐MAP+US group had similar gut microbial profiles compared with the control group (Figure S34d). A bar chart and heatmap illustrated the general microbiota composition across all groups, focusing on the family‐level abundance of gut microbiota (Figure S34e). The *Fn* group had fewer members of *Lactobacillaceae*, *Lachnospiraceae*, and *Ruminococcaceae* than the control group. The *Lachnospiraceae* family and *Ruminococcaceae* family are important butyrate‐producing bacteria in the digestive tract, and butyrate is a short‐chain fatty acid that plays an important role in anti‐tumor therapy [49, 50]. The M‐MAP+US group restored the ecological niche of *Lactobacillaceae* family, the *Lachnospiraceae* family, and *Ruminococcaceae* family. And as shown in Figure S34f, the differential bacteria among the control, *Fn*, and M‐MAP+US groups could be observed more accurately at the genus level. *Helicobacter* genus and *Streptococcus* genus, which are related to CRC pathogenicity, increased in the *Fn* group and decreased in the control and M‐MAP+US groups. However, 16S rRNA sequencing can only detect the genus level, not the species level, and studying the specific role of a certain species of bacterium in diseases is more significant. To sum up, the similarity in composition between the M‐MAP+US group and the control group suggested effective maintenance of intestinal flora balance for better therapy effectiveness.

## 3 Conclusion

This work introduces an effective approach utilizing a tumor cell membrane‐mimicking nanoparticle to excellently reverse the tumor immunosuppressive environment induced by tumor‐colonizing *F. nucleatum* and enhance the tumor therapeutic efficiency of SDT. Tumor cell membranes make nanoparticles homologous targeting, thereby improving the targeting ability and therapeutic efficiency of SDT treatment. Both in vitro and in vivo studies indicated that M‐MAP+US can effectively inflict casualties on *F. nucleatum*, reverse the tumor immune suppressive microenvironment, promote antigen presentation, and activate CTLs to enhance SDT efficiency synergistically with the immune response. While challenges remain for clinical translation, particularly in large‐scale production with consistent quality, this work powerfully articulates the strategy that addresses the complex TME complicated by bacterial co‐existence. To sum up, our work suggests a promising nanotechnology strategy with significant potential for clinical translation in delivering drugs specifically to tumors colonized with bacteria, eliminating the influence of bacteria on tumor therapy, and enhancing tumor treatment efficiency.

## Author Contributions

**Mengting Zhou**: conceptualization, investigation, methodology, data curation, visualization, writing – original draft. **Jiahao Du**: conceptualization, methodology, validation. **Cheng Li**: methodology, data curation, validation. **Yefei Zhu**: methodology. **Kairuo Wang**: validation. **Keyi Wen**: methodology. **Yuanyuan Zhang**: investigation. **Sy‐Tsong Dean Chueng**: writing – review and editing. **Qian Chen**: conceptualization, methodology, writing – review and editing, supervision, funding acquisition. **Yang Zhang**: resources, supervision. **Huanlong Qin**: funding acquisition, supervision, project administration. Mengting Zhou, Jiahao Du and Cheng Li contributed equally to this work.

## Conflicts of Interest

The authors declare no conflicts of interest.

## Ethics Statement

We promise that the study was performed according to the international, national, and institutional rules considering the animal experiments. The study protocol of animal was approved by the Animal Ethics Review Committee of Shanghai Tenth People's Hospital (SHDSYY‐2019‐2751).

## Supporting information




**Supporting File**: exp270191‐sup‐0001‐SuppMat.docx.

## Data Availability

The authors declare that all data supporting the findings of this study are available within the paper and its supplementary information files or available from the corresponding author upon reasonable request.
