# Supplementary material for: Elimination of Fusobacterium nucleatum With Biomimetic Nanoparticles to Reverse Tumor Immunosuppression and Enhance Colorectal Cancer Therapy
Source: Exploration (Beijing). 2026 Jun 26:20250551. Online ahead of print. doi: 10.1002/EXP.20250551 (PMC13394100; doi:10.1002/EXP.20250551)
Supplement: Supplementary file 1 — Supporting File: exp270191‐sup‐0001‐SuppMat.docx. [file EXP2-9999-0-s001.docx]

**Supplementary material**

**Elimination of *Fusobacterium nucleatum* with Biomimetic Nanoparticles to Reverse Tumor Immunosuppression and Enhance Colorectal Cancer Therapy**

**This file includes:**

Materials and methods

Figs. S1 to S34

Tables S1

**Materials and methods**

**Materials**

Hexadecyl trimethyl ammonium chloride (CTAC), Sodium acetate, tetraethyl orthosilicate (TEOS), 3-merraptnpropyltrimethnxysilane (MPTS), silver nitrate (AgNO_3_), 3-Aminopropyltrimethoxysilane (APTMS), Protoporphyrin IX (PpIX), 1-ethyl-3-(3-dimethylaminopropyl)carbodiimide (EDC), N-hydroxysuccinimide (NHS), 1,3-Diphenylisobenzofuran (DPBF), 2,2,6,6-Tetramethylpiperidonehydrochlorid (TEMP) Cyanine 5 (Cy5) amine, Indocyanine Green (ICG), type IV collagenase and DNase I were purchased from Sigma-Aldrich, Inc. Ammonium hydroxide (NH_3_·H_2_O) and sodium borohydride (NaBH_4_) were obtained from Sinopharm Chemical Reagent Company. Cell Counting Kit-8 (CCK-8) was sourced from Yeasen Biotechnology (Shanghai, China). The TUNEL assay kit, DCFH-DA, 4’,6-diamidino-2-phenylindole (DAPI), live/dead viability kit (Calcein-AM/PI), Membrane and Cytosol Protein Extraction Kit, Apoptosis and Necrosis Assay Kit and ATP detection kit were purchased from Beyotime Biotechnology. Brain Heart Infusion Broth medium and Columbia Blood Agar medium were purchased from Qingdao Hope Biotechnology. Matrigel was purchased by Corning, Inc. Tribromoethanol and D-Luciferin potassium were obtained from Dalian Meilun Biotechnology Co., Ltd. The bacterial DNA in the tumor tissues was extracted by QIAamp PowerFecal Pro DNA Kit (Qiagen, Hilden, Germany). Takara Biomedical Technology (Beijing) supplied the PrimeScript RT-PCR kit and PCR amplification kits. Pacific Blue anti-mouse CD45, APC anti-mouse CD3, FITC anti-mouse CD4, and APC/Fire 750 anti-mouse CD8a were purchased by BioLegend, Inc. PE anti-mouse CD11c, FITC anti-mouse CD80, BV650 anti-mouse CD86, BV605 anti-mouse CD44, PE-Cy7 anti-mouse CD62L and RB7005 anti-mouse Foxp3 were purchased by Becton, Dickinson and Company. Mouse ELISA kits for IL-2, TNF-α, and IFN-γ were purchased from ABclonal Technology Co., Ltd. Antibodies against Ki67, TLR4, MYD88, CRT, HMGB1 and HRP-conjugated Goat Anti-rabbit IgG (H+L) were obtained from Proteintech Group, Inc.

**Synthesis of M-MAP NPs**

Firstly, MSNs were synthesized according to previous literature^[1]^. In general, after hexadecyl trimethyl ammonium chloride and sodium acetate were mixed in 50 mL aqueous solution at 60 ℃ for 1 hour, 4 mL tetraethyl orthosilicate was dropwise added into the above system at 60 ℃ overnight to obtain MSN NPs. Then, to modify the sulfhydryl group (-SH) on the surface of MSNs, 150 mg MSN NPs were dissolved in 15 mL ethanol solution, and 75 µL 3-merraptnpropyltrimethnxysilane and 375 µL Ammonium hydroxide were added. Then the solution was stirred vigorously at room temperature for 12 hours. Afterward, silver nitrate (2.5 mg) was added dropwise into 5 mL of SH-MSNs aqueous solution (2 mg/mL) under stirring for 30 min. Subsequently, sodium borohydride (5 mg) was quickly added into the above solution for 1 hour and the mixture was centrifuged and washed with double distilled water three times to get Ag@MSN NPs (MA NPs). Next, to modify the amino group (-NH2) on MSNs, 160 mg MSN NPs were dissolved in 100 mL anhydrous ethanol solution, 120 µL 3-merraptnpropyltrimethnxysilane was added, and the mixture was reacted at 78 °C for 4 hours. NH2-MSNs were obtained by centrifugation. And then, adding 1.5 mg of EDC and 1.7 mg of NHS, and 8.8 mg of Protoporphyrin IX was dissolved in 3 mL of methanol and allowed to react for 30 minutes. Subsequently, 88 mg NH2-MSNs (20 mL) were added and stirred for another 12 hours. The mixture was washed alternately by water and ethanol and centrifuged to obtain Ag@MSN-PpIX NPs (MAP NPs).

The Membrane and Cytosol Protein Extraction Kit from Beyotime Biotechnology was used to isolate tumor cell membranes. CT26 tumor cells (2×10^7^-5×10^7^) were digested by pancreatic enzymes, washed 3 times with pre-cooled PBS solution and centrifuged to remove the PBS. The precipitate was further suspended in 1 mL of membrane protein extraction buffer A containing Phenylmethanesulfonyl fluoride (PMSF, final concentration 1×10^-3^ M), incubated on ice for 15 min, and then frozen and thawed three times in liquid nitrogen to rupture the cells. The cell fragments were blown off, centrifuged at 700 g for 10 min at 4℃, the upper liquid was taken, the precipitate (cell nucleus) was discarded. After centrifuging the supernatant at 14000 g for 30 minutes at 4 ℃ the liquid was discarded to isolate cell fragments. Tumor cell membranes were stored at -80 ℃.

Tumor cell membranes and MAP NPs were mixed in different ratios (5:1, 3:1, 1:1) and dispersed in ultrasound for 15 minutes. The mixture was extruded 11 times in a micro liposome extruder equipped with 400 nm polycarbonate membrane and then centrifuged at 15,000 rpm/min for 20 min to remove excess free cell membranes. And M-MAP NPs were stored at 4 ℃.

**Materials characterization**

TEM and element mapping images were obtained by Hitachi HF5000 S/TEM and JEM-2100F transmission electron microscope. Measurements of average size and zeta potential were conducted using DLS (Malvern Zetasizer Nano ZS90). UV-Vis spectrometer was measured by UV-2600 (Shimadzu, Japan). X-ray photoelectron spectroscopy (XPS) was measured by Thermo K-Alpha. Inductively coupled plasma-mass spectrometry (ICP-MS) was measured by Perkin Elmer NexION 300D.

**Encapsulation efficiency and loading capacity**

The encapsulation efficiency (EE%) of MSNs for Ag NPs or PpIX was calculated using following equation: EE (%) = Actual amount of encapsulated compound concentration (mg) / Initial amount of compound concentration (mg)

The loading capacity (LC%) of MSNs for Ag NPs or PpIX was calculated by following equation: LC (wt %) = actual amount of compound concentration (mg)/ actual amount of compound concentration (mg) + total amount of MSNs (mg)

The actual amount of Ag NPs was obtained by centrifuging MA NPs, and then detected by ICP-MS. The actual amount of PpIX was detected by UV-Vis and calculated by standard curve (Figure S1).

**Silver release profiles**

To obtain the release profiles of Ag^+^, the M-MAP NPs was dispersed and US irradiation was taken under different intensity (0.5, 1, 1.5 W cm^-2^) with prolonged durations (every 5 mins). The suspension was taken by centrifugation. The cumulative released amount of Ag^+^ was taken by ICP-MS. And the content of Ag in the suspension of MAP NPs in pH=7.4 and pH=6.4 in extended time (0, 12, 24, 48, 72 h) were detected by ICP-MS.

**^1^O_2_ detection**

The ^1^O_2_ generation via M-MAP under US was qualitatively detected by ESR and UV-vis spectra of DPBF, respectively. In detail, M-MAP (200 µg/mL) was exposed to US (1.0 MHz, 1.5 W cm^-2^, 50% duty cycle, 5 min) in the presence of TEMP (90 µM). The ESR spectrometer promptly detected the ^1^O_2_ signal, using TEMP with US for control comparison. Similarly, M-MAP (200 µg/mL) was subjected to US (1.0 MHz, 1.5 W cm^-2^, 50% duty cycle) in the presence of DPBF (40 µL, 8 mM), and the absorbance at 410 nm was recorded using a UV-vis spectroscope. The US with DPBF was used as a control. To confirm its time dependence, the mixture was subjected to US every 2 minutes.

**Cell culture**

NCM460 normal human intestinal epithelial cells, along with LLC, 4T1, and CT26 mouse cancer cells, were purchased from the American Type Culture Collection (ATCC) and grown at 37 ℃ in DMEM with 10% (v/v) FBS, 100 U/mL penicillin, and 100 µg/mL streptomycin under 5% CO_2_.

**Bacterial culture**

*Fusobacterium nucleatum* strain (ATCC 25586 obtained from ATCC) was cultured in BHI Medium under anaerobic conditions at 37 ℃ within an anaerobic chamber.

**In vitro cellular uptake**

CT26 cells were plated in 24-well plates (6×10^4^ per well) in DMEM for 24 hours. The medium was then switched to M-MAP labeled with Cy5, added at a specific time interval. After three PBS washes, nuclei were stained with DAPI. The samples were mounted and observed using LSCM. To determine the tumor homologous targeting ability of M-MAP, LLC, 4T1 and CT26 cells were plated in 24-well plates (6×10^4^ per well) in DMEM overnight. Then the medium was replaced with M-MAP labeled with Cy5. Cell types were incubated for 6 hours, washed with PBS, and stained with DAPI for LSCM imaging.

**In vitro cellular ROS detection**

CT26 cells were grown in 12-well plates (5×10^5^) in DMEM for 24 hours. Then the medium was changed with fresh PBS, MA NPs (20 µg/mL), MP NPs (PpIX: 10 µg/mL), MAP NPs (PpIX: 10 µg/mL), and M-MAP NPs (PpIX: 10 µg/mL). After coculturing 6 hours, the medium of each group was replaced by DCFH-DA in non-FBS DMEM (10 µM) and given 1 min of US (1.0 MHz, 1.5 W cm^-2^, 50% duty cycle) or without US. Following a 30-minute incubation, the cells underwent three PBS washes before being analyzed via flow cytometry.

**In vitro cell viability assay**

Flow cytometric analysis. Annexin V-FITC Apoptosis Detection Kit was used for apoptosis analysis via flow cytometry. Briefly, the processing of experimental groups was consistent with the grouping settings for ROS detection. In a dark environment, cells (1×10^6^) were resuspended in 200 µL of dye-containing binding buffer and incubated for 15 min at room temperature, then measured by flow cytometry.

Fluorescence microscope imaging. In order to visually observe the therapeutic effect, the grouping settings were the same as above. Post-US, cells were stained with calcein-AM (10 µM) and PI (15 µM) for 30 minutes in the dark to identify viable and dead cells. Finally, the images were captured using fluorescence microscopy.

CCK-8 assay. To investigate the cytotoxicity of nanocarriers without US, NCM460 cells in culture medium were seeded in 96-well plates (10^4^ cells per well) overnight. Cells were treated with MSN NPs, MA NPs, MP NPs, MAP NPs, and M-MAP NPs in different concentrations for 24 hours. Once treated, cells were washed with fresh PBS, and then 100 µL of fresh culture medium was added, followed by the addition of 10 µL of CCK-8. The absorbance at 450 nm was measured with a microplate reader after incubation at 37 ℃. To explore the influence of *F. nucleatum* on the efficacy of sonodynamic therapy (SDT), *F. nucleatum* was used to infect CT26 cells. Specifically, CT26 cells were plated into 96-well culture plates for 12 hours in non-penicillin and streptomycin medium. Then, CT26 cells were incubated with *F. nucleatum* (MOI=100:1) or without bacteria, and respectively treated with PBS, MP NPs (PpIX: 10 µg/mL), MAP NPs (PpIX: 10 µg/mL), and M-MAP NPs (PpIX: 10 µg/mL) for 6 h. After US (1.0 MHz, 1.5 W cm^-2^, 50% duty cycle, 5 min), cells washed with PBS, followed by an assessment of their viability employing the CCK-8 assay. The optical density was subsequently measured at 450 nm utilizing an automated microplate reader. To explore the cell viability of CT26 cells with or without US irradiation under different conditions, including different PpIX concentrations (0, 5.10.15 µg/mL, 1.0 MHz, 1.5 W cm^-2^, 50% duty cycle, 5 min), elevated US power densities (0, 1, 1.5, 2 W cm^-2^) and prolonged US durations (0, 1, 3, 5, 7 min), the CCK-8 assay was conducted.

**Evaluation of antibacterial properties**

To evaluate the ability of killing *F. nucleatum* by SDT, *F. nucleatum* (1×10^5^ CFU/mL) was cocultured with different treatments as following overnight, PBS, MA NPs (Ag: 10 µg/mL), MP NPs (PpIX concentration was same as MAP NPs and M-MAPs), MAP (Ag: 10 µg/mL) and M-MAP NPs (Ag: 10 µg/mL). After that, bacteria were treated with or without US for 10 min (1.0 MHz, 1.5 W cm^-2^, 50% duty cycle). The absorbance at 600 nm was measured to monitor the bacteria's growth. In addition, bacterial were treated with different concentrations of Ag NPs (0, 5, 10, 15 µg/mL) in prolonged durations (12, 24, 48 h) and OD600 was measured. The relative OD600 value was corrected by subtracting its initial value since each component in M-MAP NPs also contribute to the OD600. Meanwhile, the suspensions were serially diluted and plated on Columbia blood agar plates and cultured in an anaerobic chamber for 48 hours. The number of colonies were counted.

LCSM imaging. To visually observe the therapeutic effect, the treatments were the same as the above antibacterial assay. After the US, 100 µL of *F. nucleatum* suspensions were incubated with 1 µL of live & dead bacterial staining solution containing DMAO (live & dead bacteria) and EthD-Ⅲ (dead bacteria) in darkness at ambient temperature for 15 minutes. After that, 5 μL of the stained bacterial suspensions was placed on a glass slide, and *F. nucleatum* was observed by LCSM.

**In vitro direct DC activation**

BMDCs were isolated from the bone marrow of BALB/c mice using established protocols. Femurs were harvested from healthy, 4-week-old male BALB/c mice. The bone marrow was extracted by carefully flushing the femur and tibia with RPMI 1640 culture medium after both ends of the bones were trimmed. The extracted bone marrow was then passed through a 70 µm cell strainer into a centrifuge tube and centrifuged at 150 g for 10 minutes, followed by red blood cell lysis. The resulting cell pellet was resuspended in 12 mL of RPMI 1640 culture medium, supplemented with 20 ng/mL granulocyte-macrophage colony-stimulating factor (GM-CSF), and seeded into 6-well plates. The cultures were maintained at 37 ℃ in a 5% CO_2_ atmosphere. On day three, an additional 12 mL of fresh culture medium containing 20 ng/mL GM-CSF was added, with 2 mL distributed to each well, and incubation continued for an additional three days. BMDCs were subsequently obtained by collecting the non-adherent and loosely adherent cells from the culture.

To study in vitro DC activation, CT26 cells, *F. nucleatum,* and BMDCs were co-cultured using a transwell system. CT26 cells, *F. nucleatum,* and CT26 cells with *F. nucleatum* (MOI=100:1) were seeded respectively in the upper compartment and treated with M-MAP NPs for 6 h, followed by US (1.0 MHz, 1.5 W cm^-2^, 50% duty cycle, 5 min). Subsequently, BMDCs were cultured in the lower chamber overnight. The cells were then stained with anti-CD11c, anti-CD80, and anti-CD86 antibodies for 30 min. Following staining, the cells were washed and analyzed using flow cytometry.

**Tumor model establishment**

CT26/*Fn* CRC subcutaneous model establishment. CT26 cells were co-cultured with *F. nucleatum* (cell intensity: 5×10^6^, MOI=100:1) for 6 hours. After that, the mixture (100 µL) was subcutaneously injected into each male BALB/c mouse (6 weeks old) to construct the CT26/*Fn* colorectal subcutaneous model.

In situ CT26-luc/*Fn* tumor model of CRC establishment. CT26-luc cells were co-cultured with *F. nucleatum* (MOI=100:1) for 6 hours. Tribromoethanol was used to anesthetize 6-week-old male BALB/c mice. The fur on the abdomen of each mouse was taken off with an animal electric razor. After disinfecting the surgical area of the abdomen with povidone Iodine three times, an abdominal incision was created and the caecum was exteriorized. Then, CT26-Luc cells with *F. nucleatum* or without bacteria (cell intensity: 2×10^6^) in 50 µL of normal saline solution with Matrigel (1/1, v/v) were inoculated into the caecum subserosa. The caecum was inserted back into the peritoneal cavity, followed by closing the abdominal wall and skin.

CT26 bilateral tumor model establishment. CT26 cells were co-culture with *F. nucleatum* as mentioned. After that, the mixture (100 µL) was subcutaneously injected into each male BALB/c mouse for one side. After 9 days, the CT26 cells without bacteria were subcutaneously injected to another side of mouse.

**In vivo toxicity assay**

For in vivo toxicity evaluation, the six-week-old male BALB/c mice were randomly assigned to four distinct groups. After being injected with saline, MP NPs, MAP NPs, and M-MAP NPs (the PpIX dose of 10 mg/kg, 100 µL) for 14 days, the weight of mice was recorded every two days. And the mice were euthanized to obtain the main organs and blood samples for the blood biochemical and histological examination.

**In vivo biodistribution and fluorescence imaging**

CT26 subcutaneous tumor mice were injected with ICG-labeled M-MAP NPs (the PpIX dose of 10 mg/kg, 100 µL) by tail vein at different time points (0, 12, 24, 48, 72 hours). At set times, the mice were sacrificed, and their primary organs (heart, liver, spleen, lung, kidney) and tumors were gathered. The major organs and tumors were imaged under IVIS. In order to observe the sustained accumulation of Ag NPs in tumor tissues, ICP-MS was used to detect Ag content in tumor sites at various time points.

To determine the tumor homologous targeting ability of M-MAP NPs, LLC (C57BL/6), 4T1 (BALB/c), and CT26 (BALB/c) subcutaneous tumor mice were established. Each group of mice was injected with ICG-labeled M-MAP NPs (the PpIX dose of 10 mg/kg, 100 µL) by the tail vein. After 24 hours, the mice were sacrificed, and their main organs and tumors were gathered for examination with an IVIS in vivo imaging system.

**In vivo anti-cancer therapy**

CT26/*Fn* CRC subcutaneous model. Once established, the mice with tumors were randomly divided to six distinct groups: control (without *Fn*), *Fn*, MP NPs, MAP NPs, M-MAP NPs (PpIX: 10 mg/kg, 100 µL; US: 1.0 MHz, 1.5 W cm^-2^, 50% duty cycle, 10 min) and M-MAP NPs without US. On days 0, 3, and 6, the different groups of mice received injections of specific formulations through tail vein. And mice were exposed to US after intravenous injection of nanoparticles after 24 hours. Measurements of tumor volume and mice's body weight were taken every other day. Volume was determined with the formula: (tumor width)^2 × (tumor length)/2. Mice were euthanized and their tumors were dissected after 14 days of treatment. For histopathological analysis, H&E, Ki-67, and TUNEL were used for tumor sections staining.

In situ CT26-luc/*Fn* tumor model of CRC. After establishment, tumor-bearing mice were randomly divided into six groups, like a subcutaneous tumor model. And the treatments were the same as above. The whole mouse body tumor burden was monitored with bioluminescence radiance intensity using AniView animal live imaging system (BLT Photon Technology) after the mice were injected intraperitoneally with 150 mg/kg D-Luciferin and quantified as the luminescence radiance intensity (p/s/cm^2^/sr) using AniView software. After 14 days of treatment, the mice were euthanized and their gastrointestinal tracts were dissected. The weight and volume of the tumor were measured. The tumor sections of mice were stained with H&E, Ki-67and TUNEL for histopathological analysis.

Bilateral CT26 tumor model. After establishment, tumor volume was taken every other day. Volume was determined with the formula: (tumor width)^2 × (tumor length)/2. Mice were euthanized and their tumors were dissected after 14 days of treatment. For histopathological analysis, H&E, Ki-67, TLR4 and MYD88 were used for tumor sections staining. For immune response detection, the distal tumors and spleen were taken for flow cytometry analysis.

**The detection of bacterial abundance of *F. nucleatum* in the tumor**

For the detection of *F. nucleatum* abundance in tumors, a QIAamp PowerFecal Pro DNA kit was used. The extraction of bacterial DNA was done following the manufacturer's guidelines, and the concentrations were detected using a NanoDrop2000 spectrophotometer. The primers were synthesized by BGI Genomics Co., Ltd. (Table S1). The real-time quantitative PCR program was performed following the manufacturer’s instructions. Values were calculated through the 2^–ΔΔCT^ method, using GAPDH as a housekeeping gene. The fluorescence in situ hybridization (FISH) was used to detect the colonization of *F. nucleatum* in tumor sites.

**In vivo flow cytometry analysis**

Following a 14-day period of varied treatments, each cohort of mice was euthanized, and both tumor tissues and spleens were harvested. The tumor tissues were subjected to enzymatic digestion at 37 ℃ using type IV collagenase and DNase I for a duration of 2 hours. Subsequently, the digested tumor tissues were passed through a 70 µm cell strainer to isolate single-cell suspensions. For the spleen, the tissues were ground directly. All the red cells in the tumor and spleen were removed by ACK lysis. For the subsequent staining of various antibodies, the cells underwent three washes using a cell staining buffer.

Single-cell suspensions obtained from the tumor were incubated with various kinds of antibodies (anti-mouse CD45, anti-mouse CD3, anti-mouse CD4, anti-mouse CD8) for 30 min in the dark. The obtained single-cell suspensions from the spleen were stained with anti-mouse CD45, anti-mouse CD11c, anti-mouse CD80, and anti-mouse CD86 for 30 min in the dark. Flow cytometry analysis was conducted on the cells after three times washing. The gating strategy was shown in Figure S33.

**In vivo cytokine detection**

All mice were euthanized, and peripheral blood was drawn from the eyeball 14 days following the initial treatment. ELISA kits were employed to quantify the concentrations of cytokines, specifically IL-2, TNF-α, and IFN-γ, in murine serum samples.

**In vitro evaluation of immunogenic cell death**

Immunofluorescence detection showed the expression of CRT and HMGB1. CT26 cells were seeded into a 24-well plate overnight. After that, M-MAP NPs and *F. nucleatum* with M-MAP NPs were treated with CT26 cells for 6 hours. US irradiation was taken (1.0 MHz, 1.5 W cm^-2^, 50% duty cycle, 5 min) and the cells were treated as previous mentioned^[2]^ for immunofluorescence detection. And the cell culture supernatant was taken for ATP detection by commercialized kit.

**Bulk RNA sequencing**

Total RNA was extracted utilizing a TRIzol total RNA extraction kit (TIANGEN). The isolated RNA was subsequently employed for library construction and sequencing. Following library construction, the quality of the library was evaluated with Agilent 2100 Bioanalyzer (Agilent, USA). The library was then sequenced on the Illumina NovaSeq 6000 platform for raw reads generation.

Differential expression analysis of genes was conducted using DESeq2 software (version R3.6.2) to compare two distinct groups. Genes were deemed differentially expressed if they met the criteria of a false discovery rate (FDR) below 0.05 and an absolute fold change of ≥ 2. Gene set enrichment analysis (GSEA) was performed using the function available in the clusterProfiler package, with the gene list sorted by log2 fold-change. The differentially expressed genes were subsequently analyzed for enrichment in GO functions and KEGG pathways.

**16S rRNA sequencing**

In order to detect the influence of intravenous injection of M-MAP NPs on the intestinal flora of mice, the CT26/*Fn* subcutaneous tumor mice were established. All mice were divided randomly into three groups (control, *Fn,* and M-MAP+US). On day 14, fecal samples from each mouse were gathered in autoclaved sterile microcentrifuge tubes and stored at -20 ℃. Fecal samples were shipped to Shanghai Bioprofile Technology Co., Ltd. for analysis. PCR products were sequenced through the Illumina MiSeq platform and analyzed by Shanghai Bioprofile Technology Co., Ltd.

**Statistical analysis**

Results were presented as mean values accompanied by the standard deviation (SD). Statistical analyses were performed using GraphPad Prism. For comparisons involving more than two groups, a one-way analysis of variance was employed, while a student's t-test was utilized for comparing two groups. In each case, statistical significance was denoted as follows: * P < 0.05, ** P < 0.01, *** P < 0.001, with "ns" indicating a lack of significance.


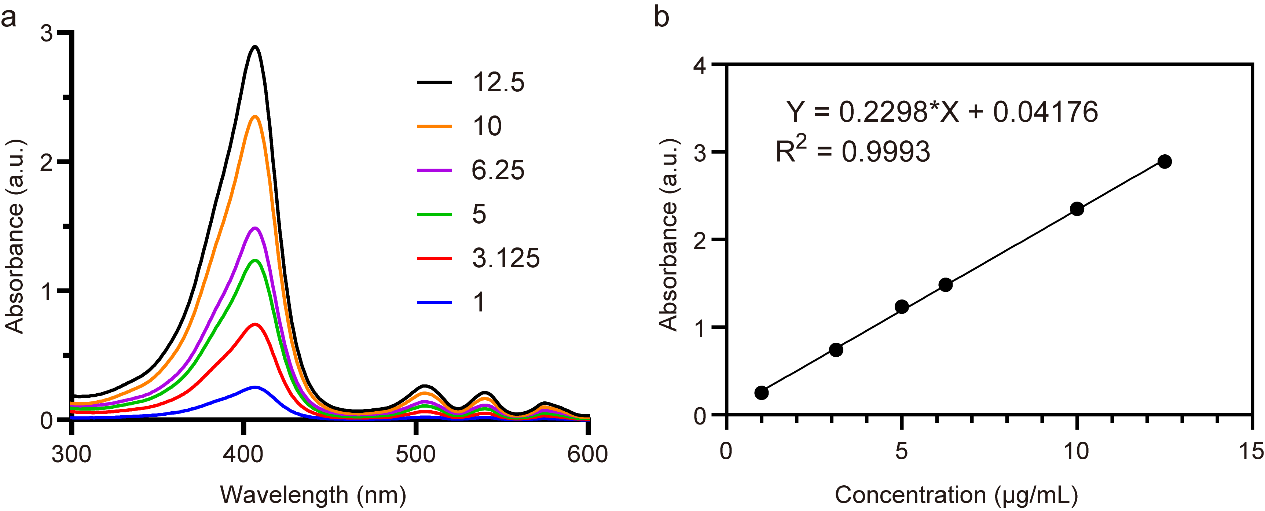


**Figure S1**: (a) UV-vis-NIR spectra of protoporphyrin at various concentrations (1, 3.125, 5, 6.25, 10, 12.5 µg/mL). (b) Absorbance plot of protoporphyrin across different concentrations.


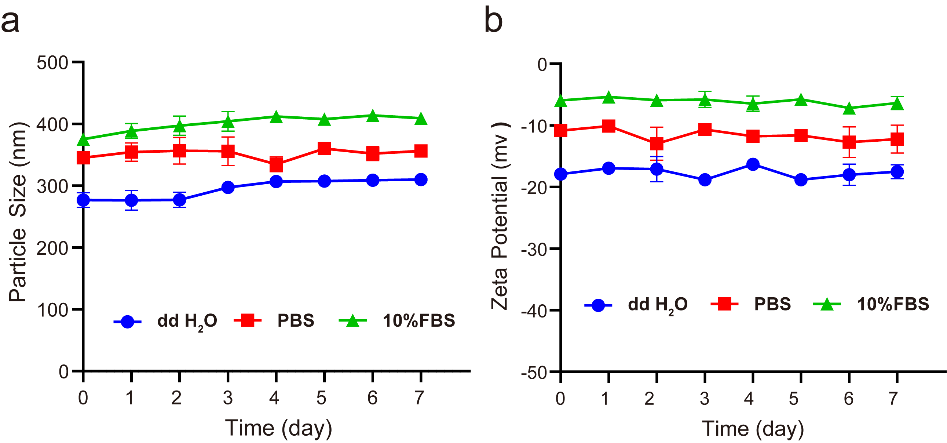


**Figure S2**: (a) Dynamic light scattering and (b) zeta potential of M-MAPs in deionized water, PBS and 10%FBS in DMEM medium for seven days. Data are presented as means ± SD (n=3).


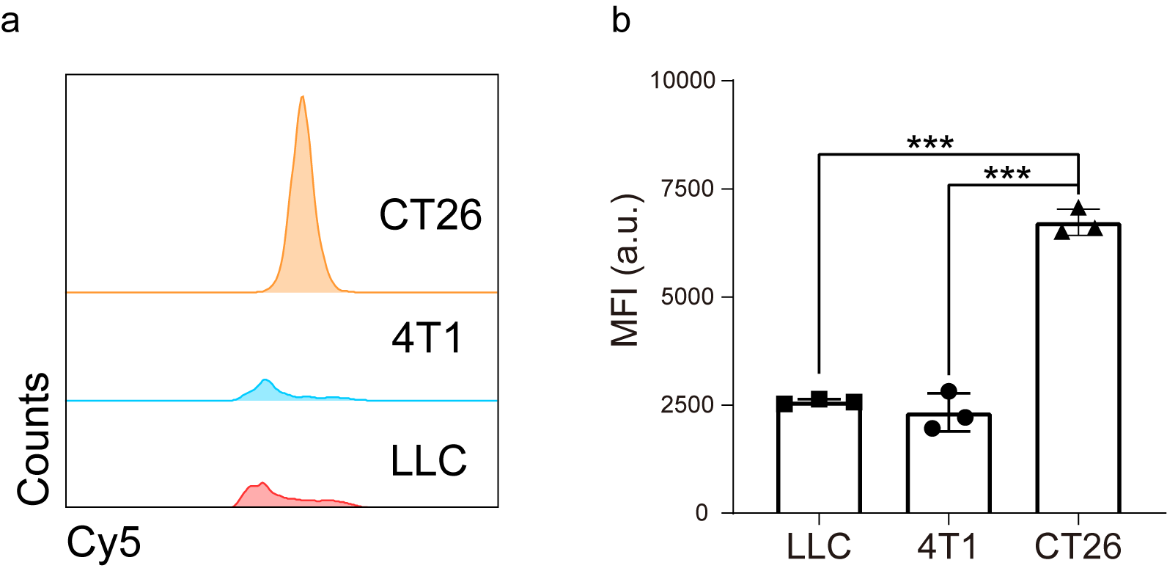


**Figure S3**: (a) LLC, 4T1 and CT26 cells were incubated with M-MAP-Cy5 for 6h for flow cytometry measurements. (b) Statistical analysis of the Cy5 intensity in LLC, 4T1 and CT26 cells after incubation with M-MAP-Cy5. Data are presented as the means ± SD (n=3). *** P < 0.001.


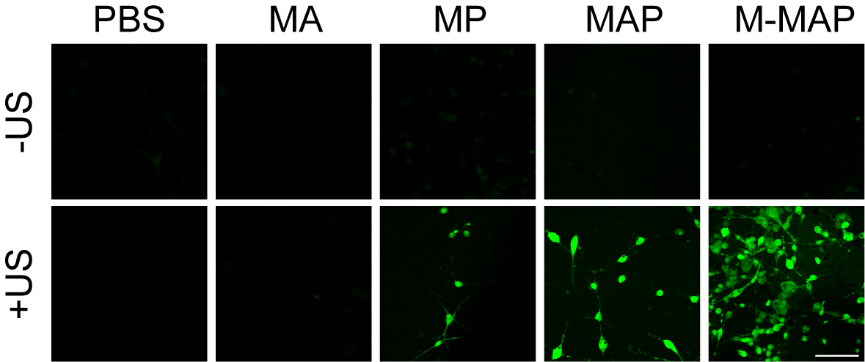


**Figure S4**: Representative LSCM micrographs of ROS levels of CT26 cells after different treatments (scale bar, 50 µm).


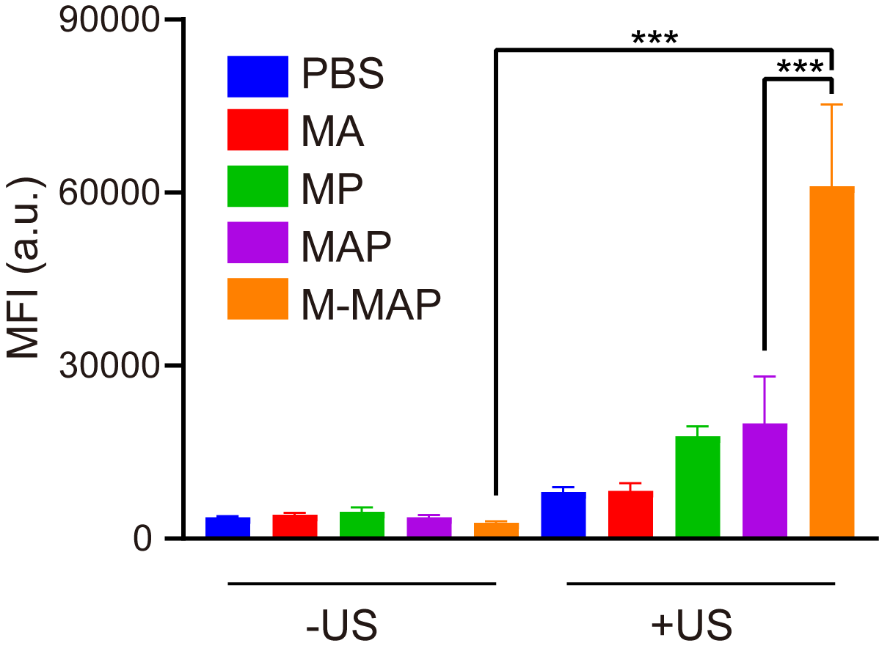


**Figure S5**: Quantitative analysis of ROS fluorescence intensity in CT26 cells of each group. Data are presented as the means ± SD (n=3). *** P < 0.001.


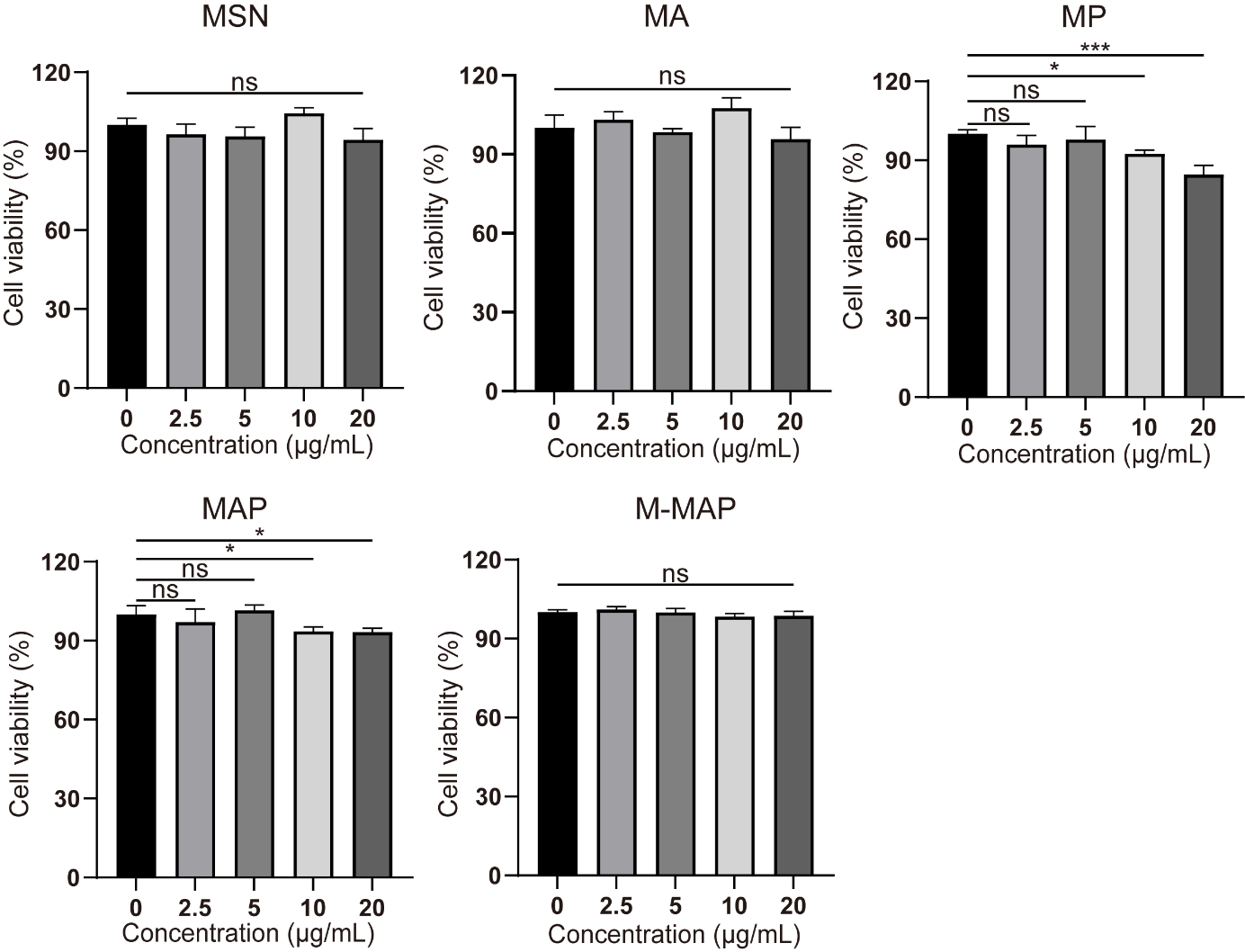


**Figure S6**: Cell viability of NCM460 incubated with different formulations (MSN NPs, MA NPs, MP NPs, MAP NPs and M-MAP NPs) of various concentrations for 24 h. Data are presented as means ± SD (n=4). ns, not significant.


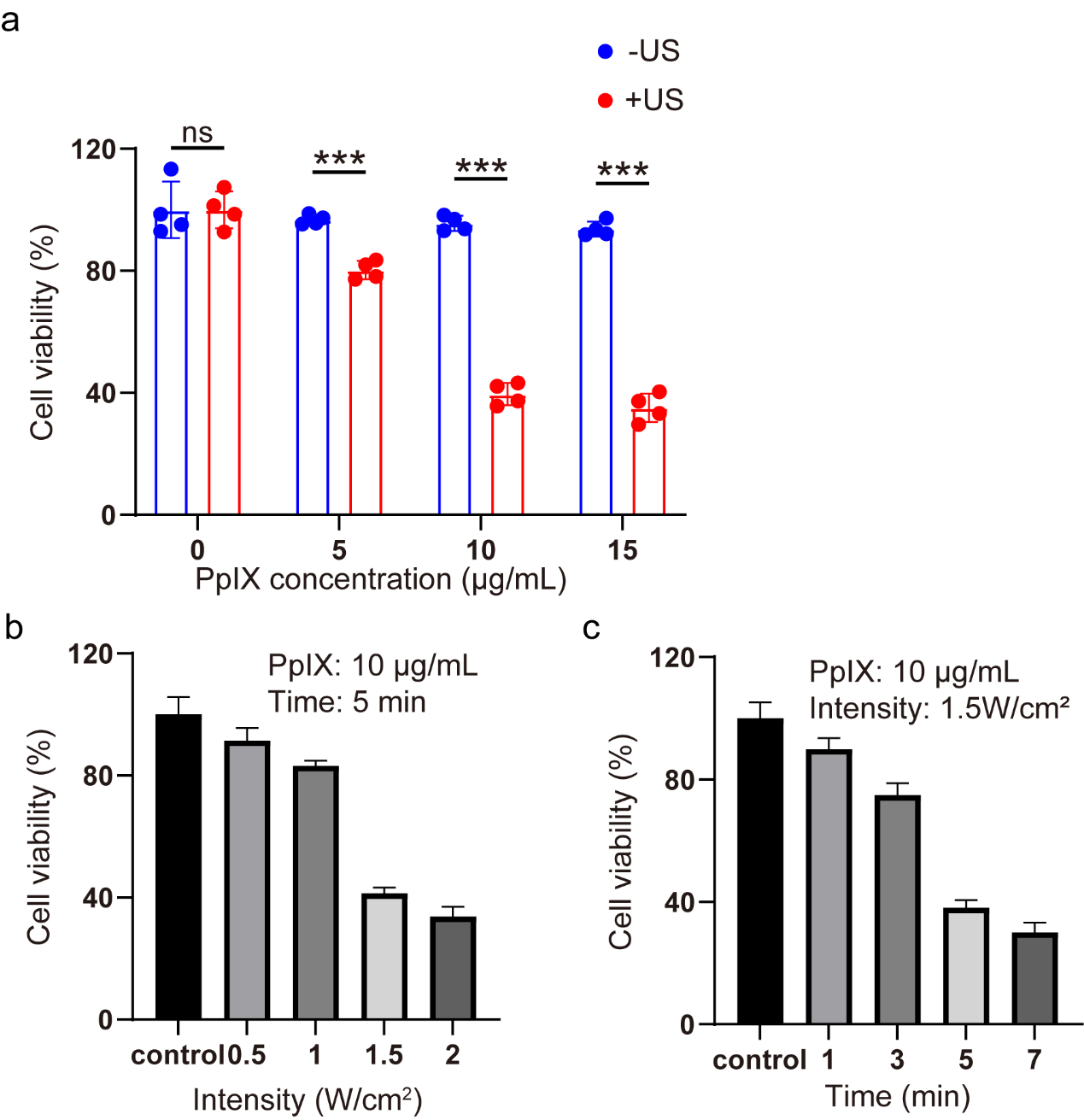


**Figure S7**: Cell viability of CT26 with or without US irradiation under different conditions, including different PpIX concentrations (a), elevated US power densities (b) and prolonged US durations (c). Data are presented as means ± SD (n=4). ns, not significant. *** P < 0.001.


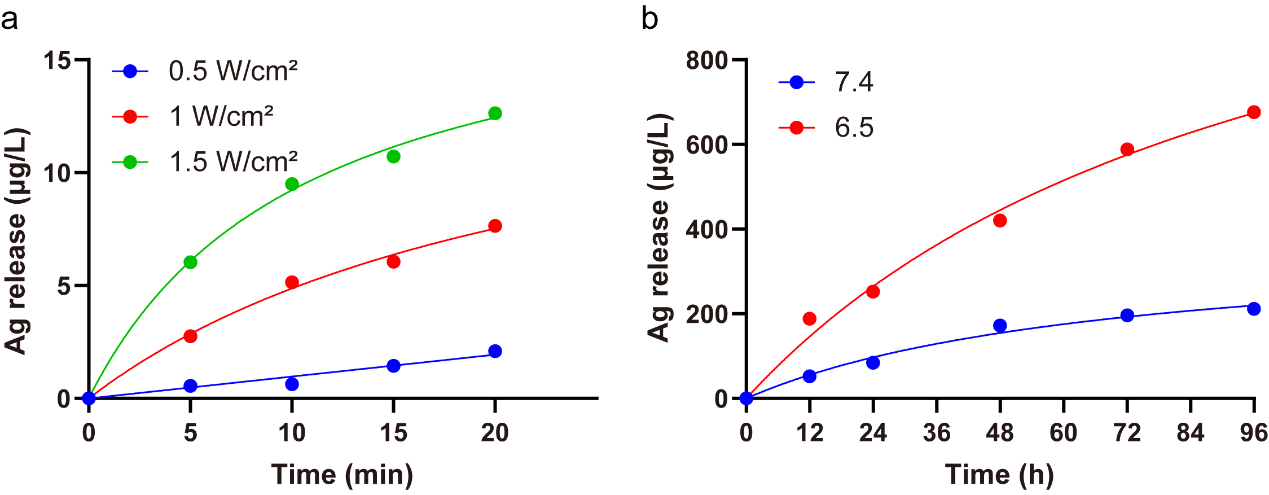


**Figure S8**: Silver ion release profiles of M-MAP NPs under different US intensity (a) and in different pH(b) with prolonged durations.


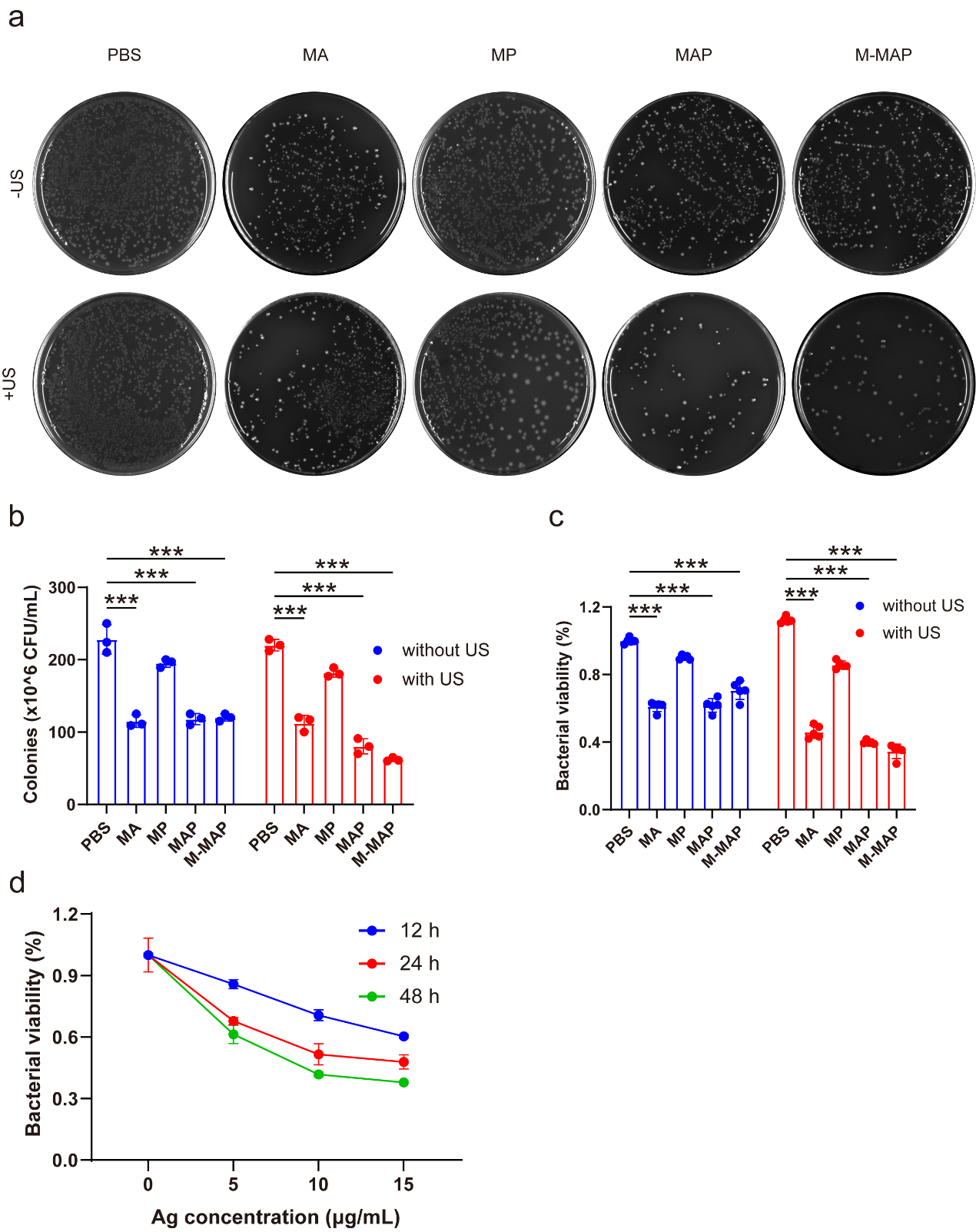


**Figure S9**: (a) Representative photographs of *F. nucleatum* which were treated by different groups (US irradiation, 1.0 MHz, 1.5 W cm^-2^, 50% duty cycle,10 min) and then coated the bacteria on Columbia blood agar plates in the anaerobic chamber for 48 h. (b, c) The viability of *F. nucleatum* under various treatments was determined by plate counting (n=3) and OD600 (n=5). (d) The viability of *F. nucleatum* with different Ag concentration in prolonged duration (n=5). Data are presented as means ± SD. *** P < 0.001.


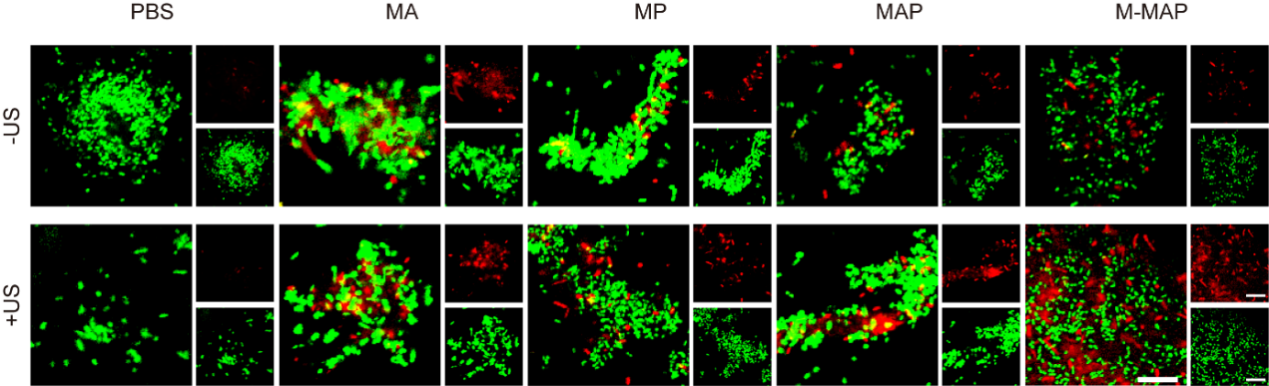


**Figure S10**: CLSM images of live/dead bacteria stained with DMAO/EthD III after various treatments which were same as the OD600 assay.


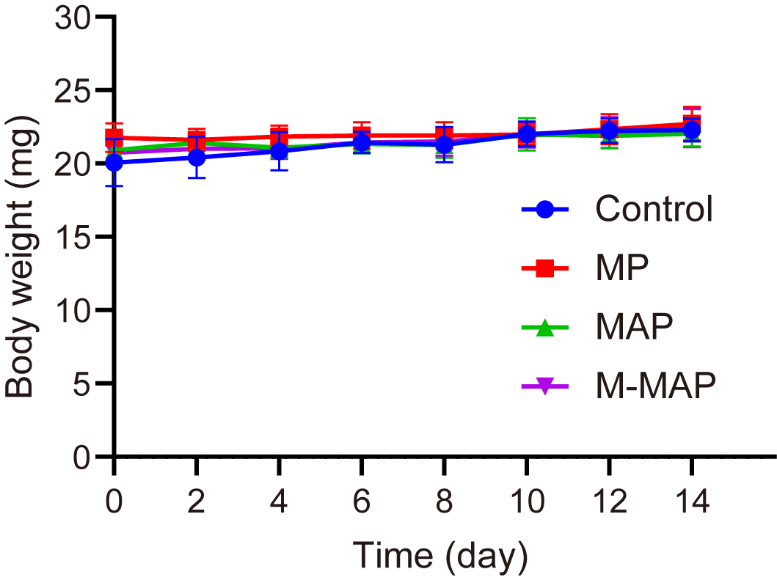


**Figure S11**: Curves of mice body weight of four groups, including intravenously injected with saline, MP NPs, MAP NPs and M-MAP NPs (the PpIX dose of 10 mg/kg, 100 µL) for 14 days. Data are presented as means ± SD (n=5).


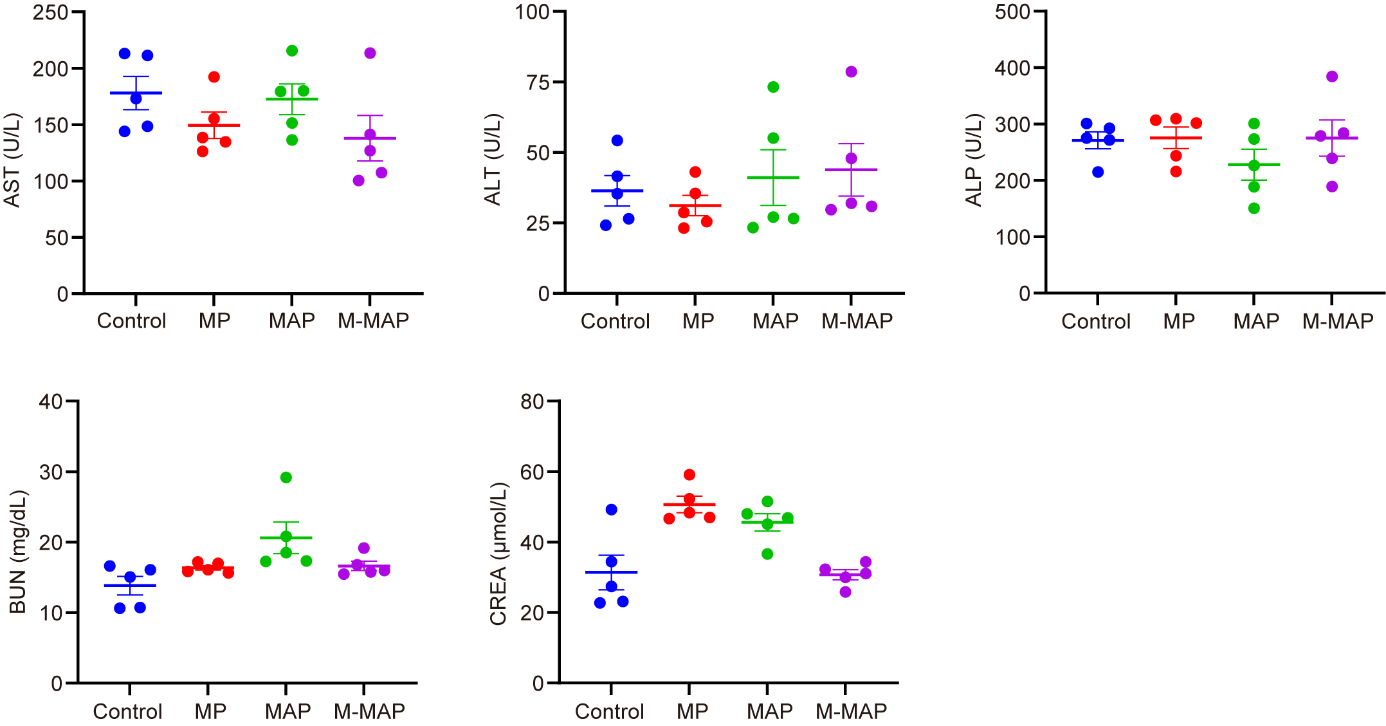


**Figure S12**: Examination of AST, ALT, ALP, BUN, and CREA levels in the serum of BALB/c mice following intravenous injection of saline, MP NPs, MAP NPs and M-MAP NPs (the PpIX dose of 10 mg/kg, 100 µL) for 14 days. Data are presented as means ± SD (n=5).


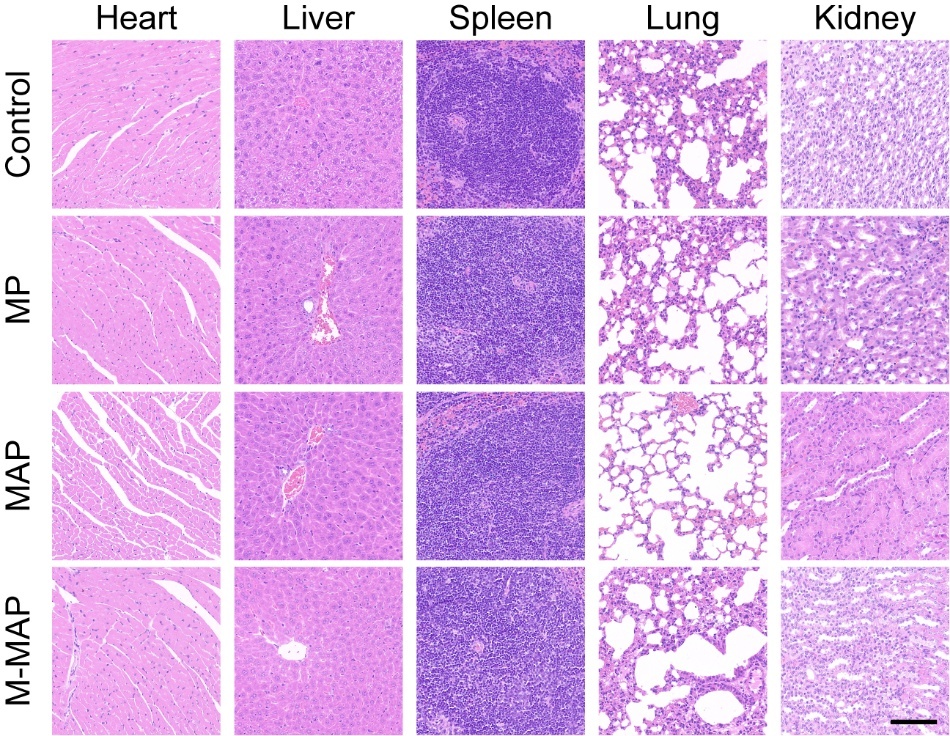


**Figure S13**: Microscopic images of primary organ sections following intravenous administration of various formulations (bar scale, 20 µm). Mice were injected with saline, MP NPs, MAP NPs and M-MAP NPs (the PpIX dose of 10 mg/kg, 100 µL) for 14 days.


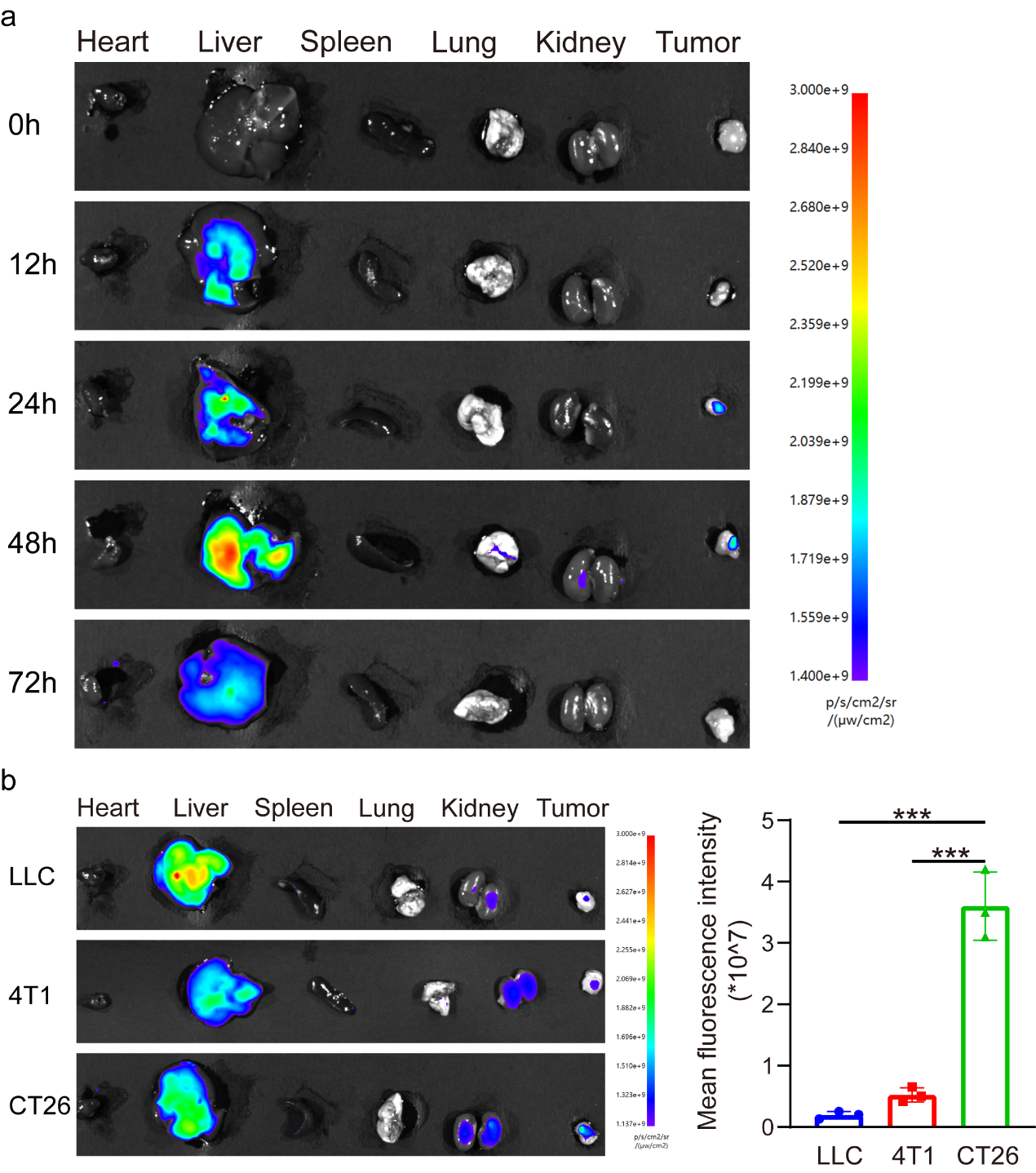


**Figure S14**: (a) In vivo distribution and fluorescence imaging of mice administered M-MAPs through the tail vein at various times (0, 12, 24, 48, 72 h). (b) Fluorescence photographs of tumor homologous targeting ability of M-MAP NPs, which 4T1 and LLC tumor models were used as control. And mean fluorescence intensity of tumor in different groups. Data are presented as means ± SD (n=3). *** P < 0.001.


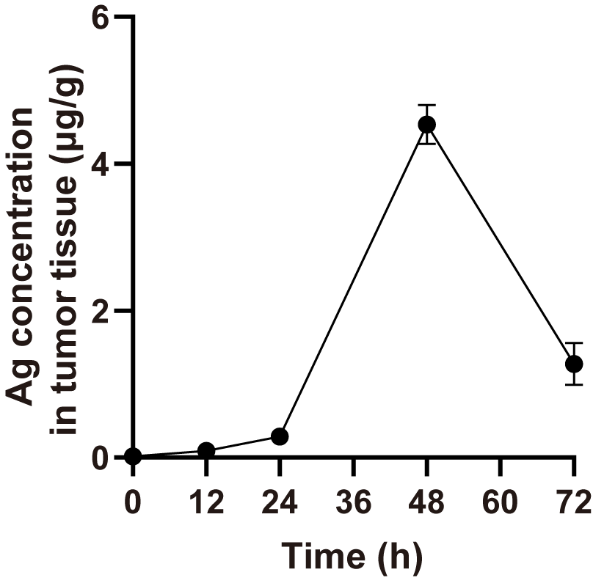


**Figure S15**: Profiles of Ag NPs accumulation into CT26 tumor-bearing mouse at predetermined time points after tail vein injection of M-MAP NPs (0 h, 12 h, 24 h, 48 h, 72 h).


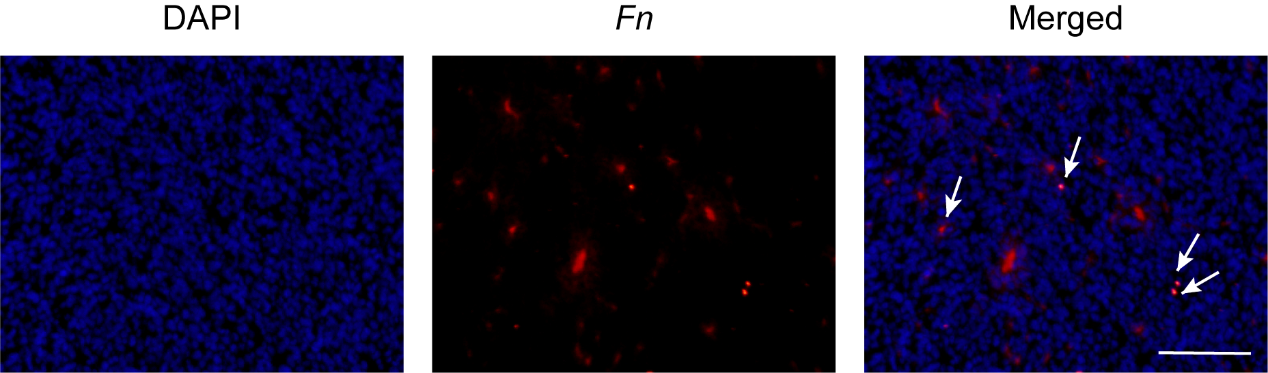


**Figure S16**: Representative photos of fluorescence in situ hybridization (FISH) for proving the colonization of *Fn* (red, with white arrows) in tumor tissue (blue for nucleic acid). Bar=100 µm.


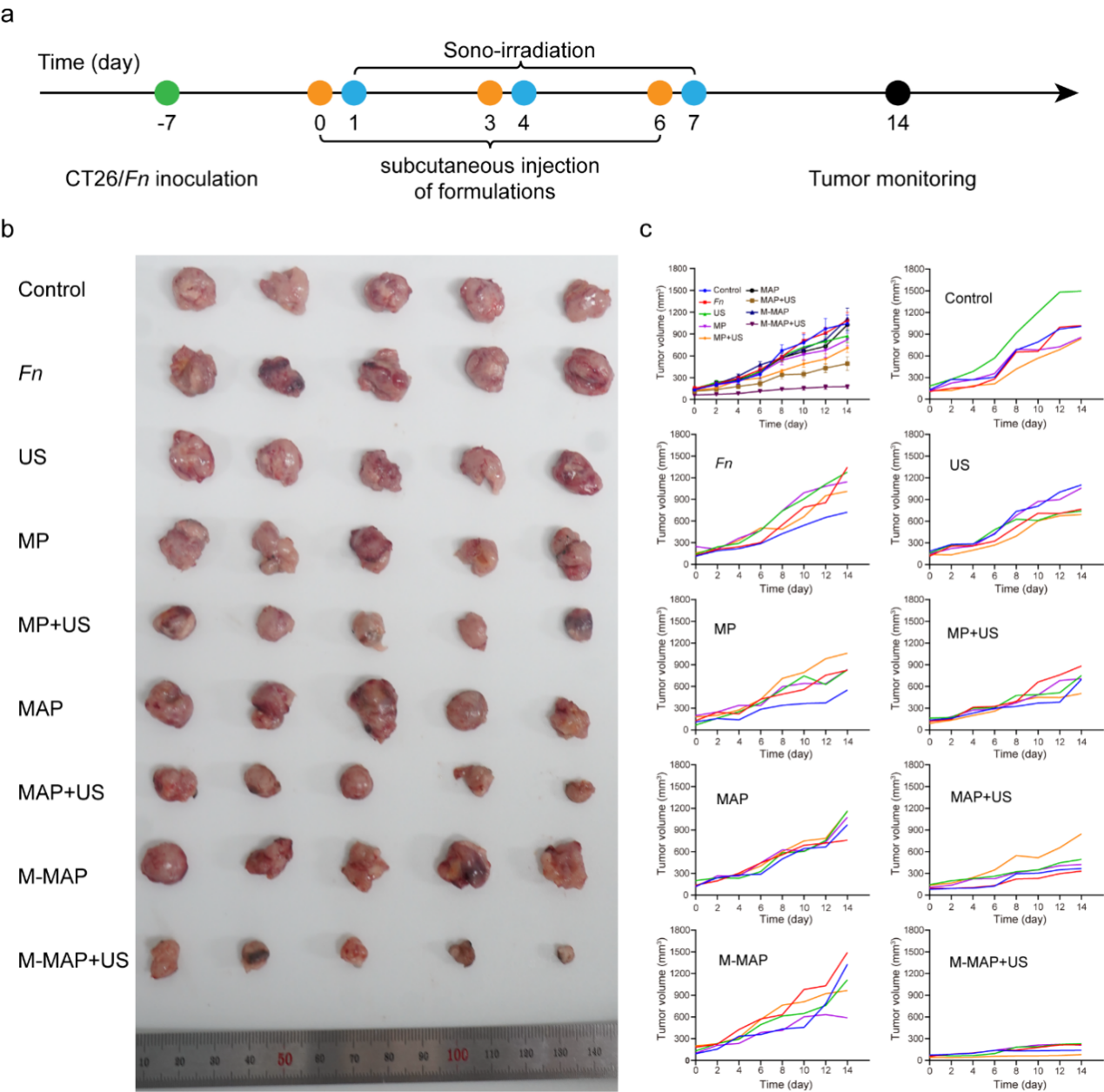


**Figure S17**: Subcutaneous administration of M-MAP NPs with SDT successfully hindered the growth of CT26/*Fn* subcutaneous tumors. (a) Schematic depiction of mice with tumors using multiple formulations (saline, MP NPs, MAP NPs and M-MAP NPs) under US irradiation (1.0 MHz, 2.5 W cm^-2^, 50% duty cycle, 10 min). (b) Digital photos of excised tumors from different groups of mice on the 15th day. (c) Tumor-volume evolutions in different groups (all and separately) during the therapeutic period. Data are presented as means ± SD (n=5).


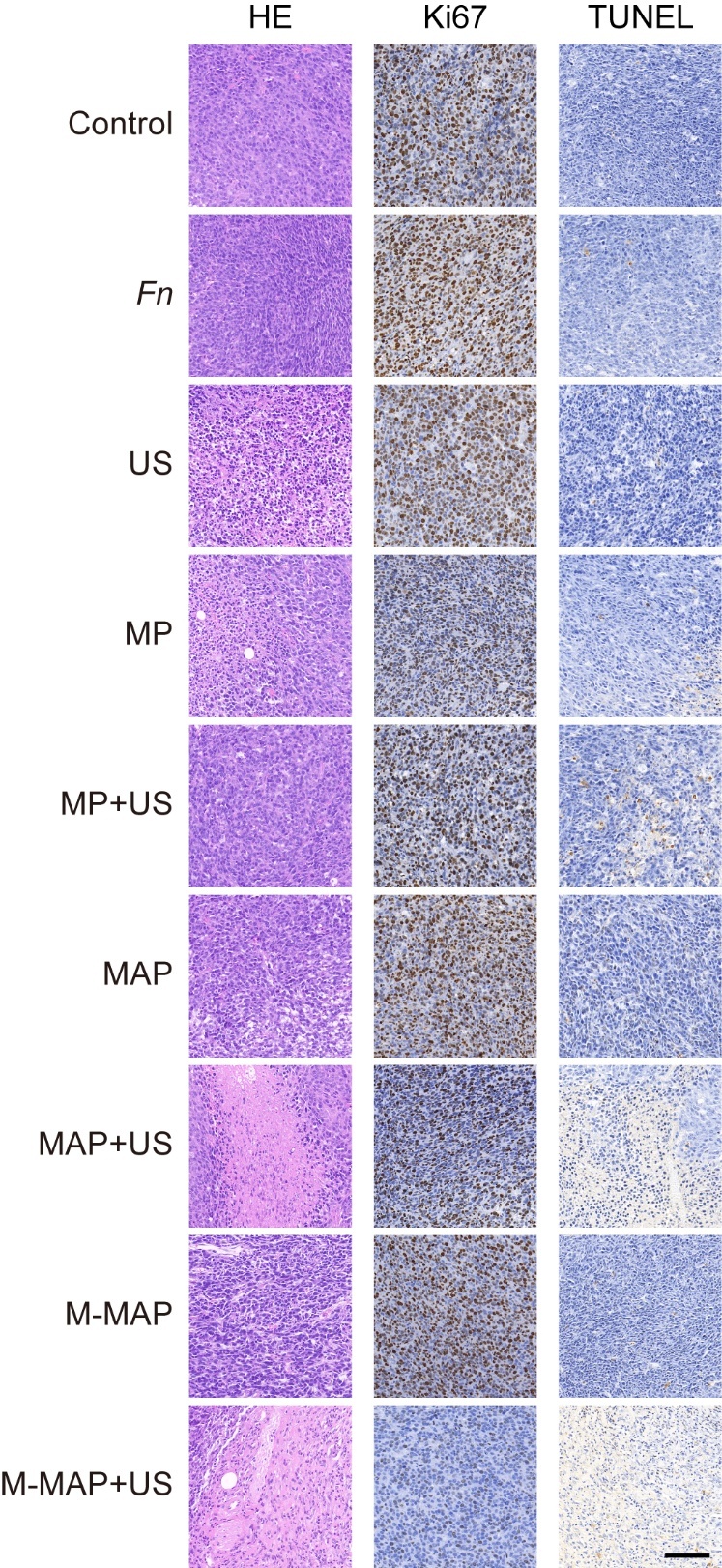


**Figure S18**: Representative photographs of H&E, Ki67 and TUNEL staining of excised tumor sections of different groups (bar=100 µm).


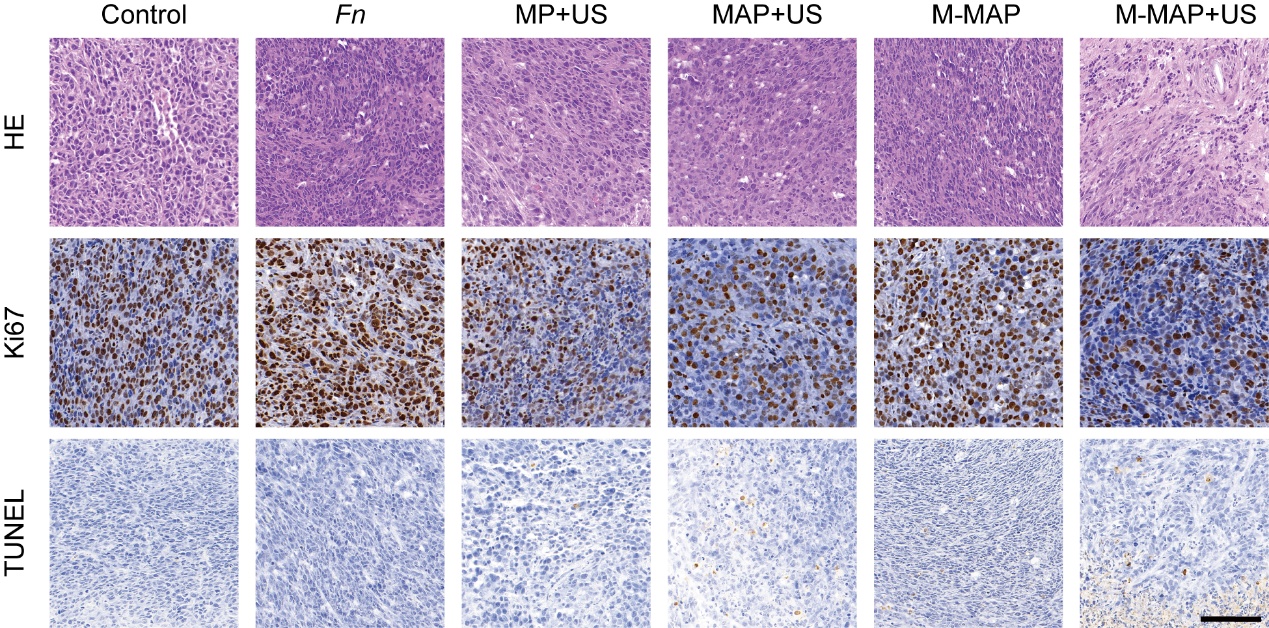
**Figure S19**: Representative photographs of H&E, Ki67, and TUNEL staining of excised tumors of in situ CT26-luc/*Fn* tumor model in different groups. (bar=100 µm).


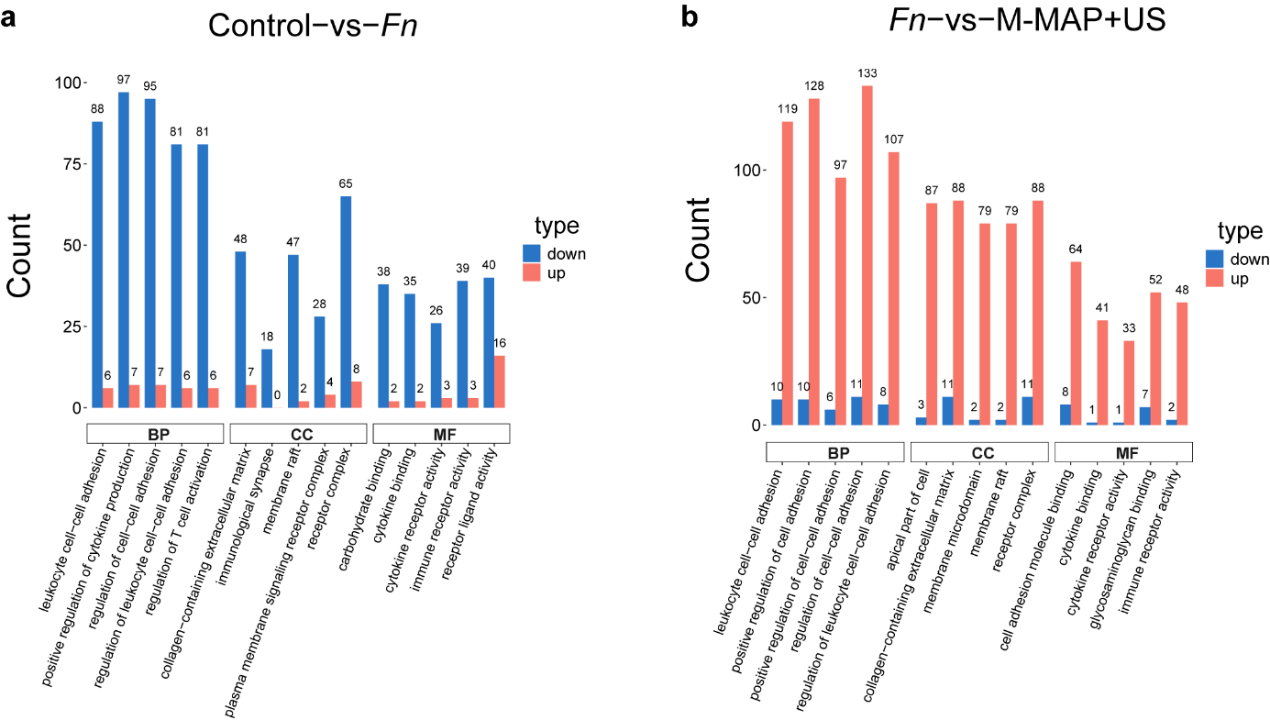


**Figure S20**: In GO enrichment analysis results, the most significant top 5 terms in (a) control vs *Fn* and (b) *Fn* vs M-MAP+US. The horizontal axis of the chart is GO Term (Biological Process, BP; Cellular Components, CC; Molecular Function, MF), and the vertical axis is count. Orange represents the upregulated genes enriched in this pathway, blue represents the downregulated genes enriched in this pathway, the numbers on the chart represent the specific number of genes.


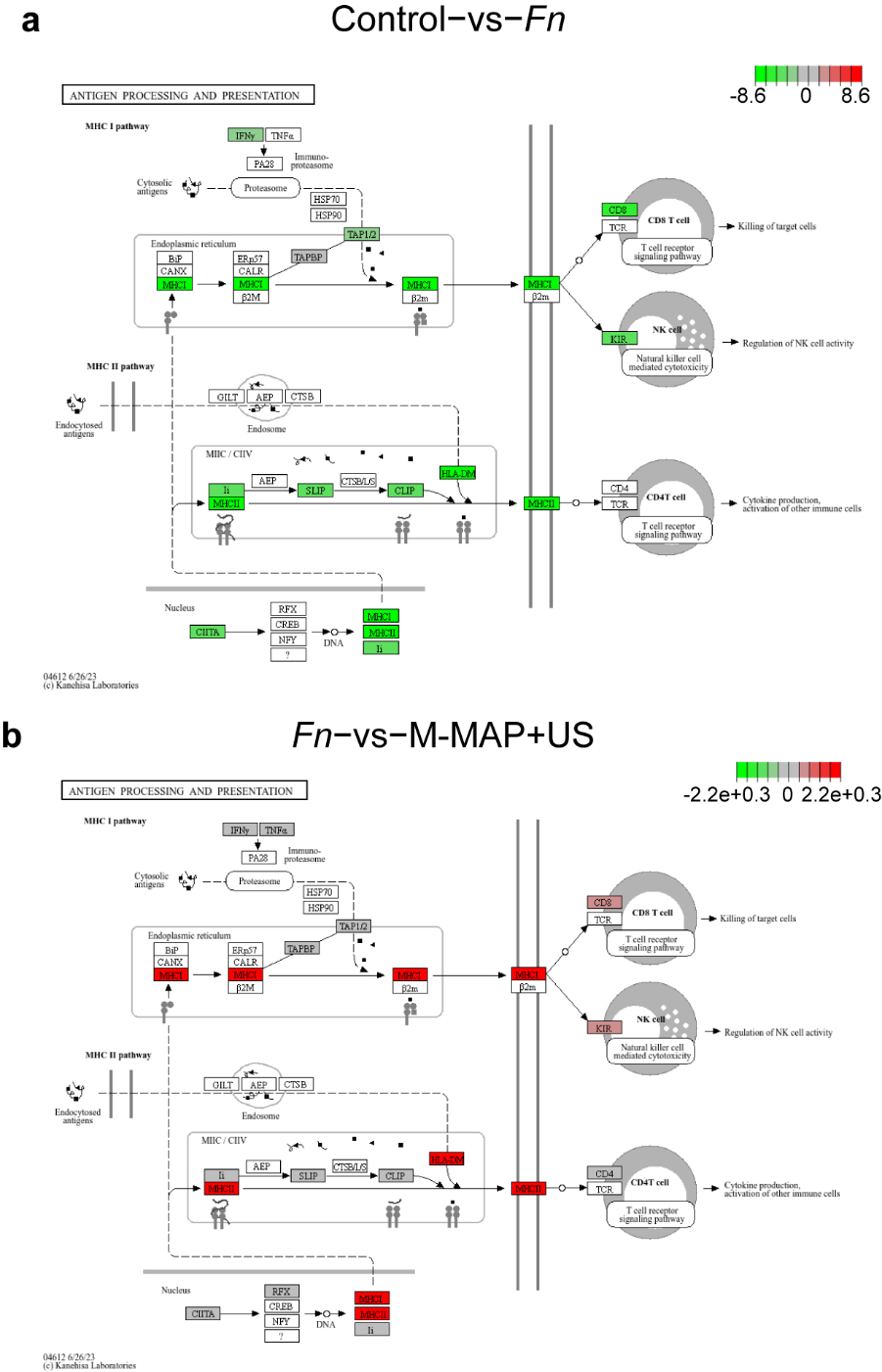


**Figure S21**: From the KEGG enrichment analysis results, antigen processing and presentation pathway were selected in (a) control vs *Fn* and (b) *Fn* vs M-MAP+US. The colors on the graph represent the differentially changing genes on this pathway. The genes marked with colors in the figure, the redder the color, the greater the upward adjustment; The greener the color, the greater the downregulation. The pathway map is from the KEGG database (Pathway ID: 04612).^[3]^ https://www.kegg.jp


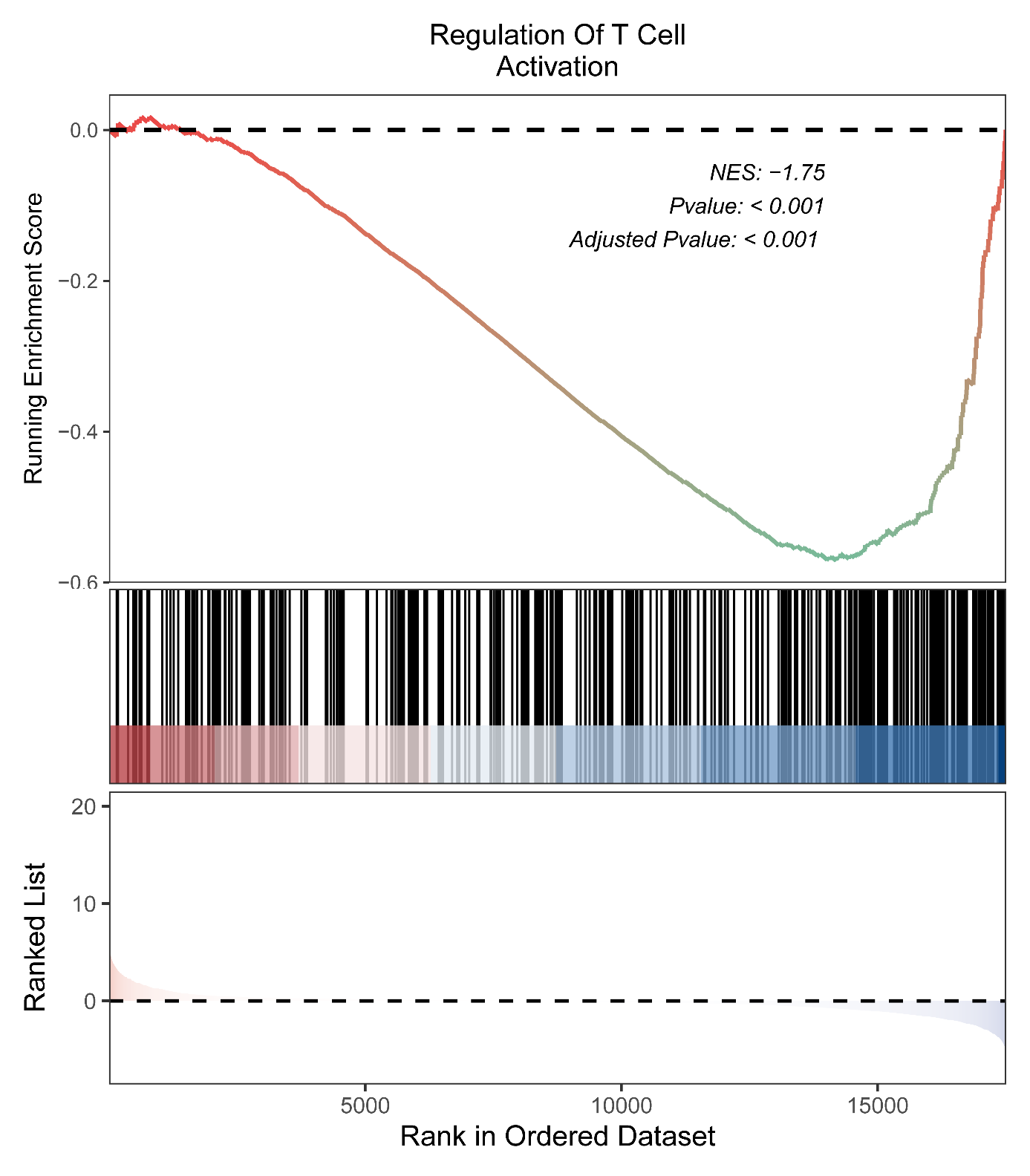


**Figure S22**: The significantly down-regulated enrichment gene sets by Gene Ontology Enrichment Analysis (GSEA) about regulation of the T cell activation through GO enrichment analysis (control vs *Fn*).


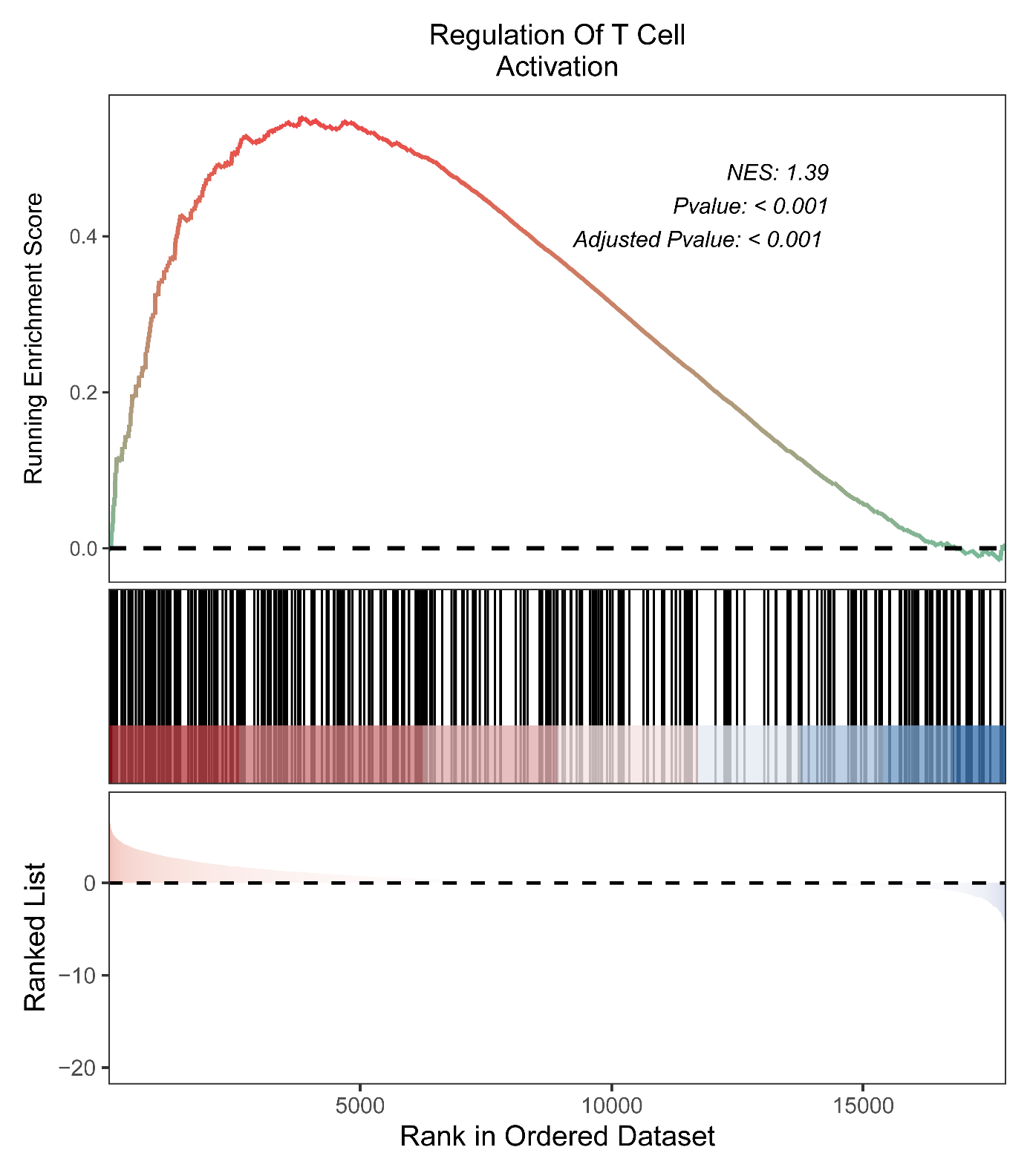


**Figure S23**: The significantly up-regulated enrichment gene sets by GSEA about the regulation of T cell activation through GO enrichment analysis (*Fn* vs M-MAP+US).


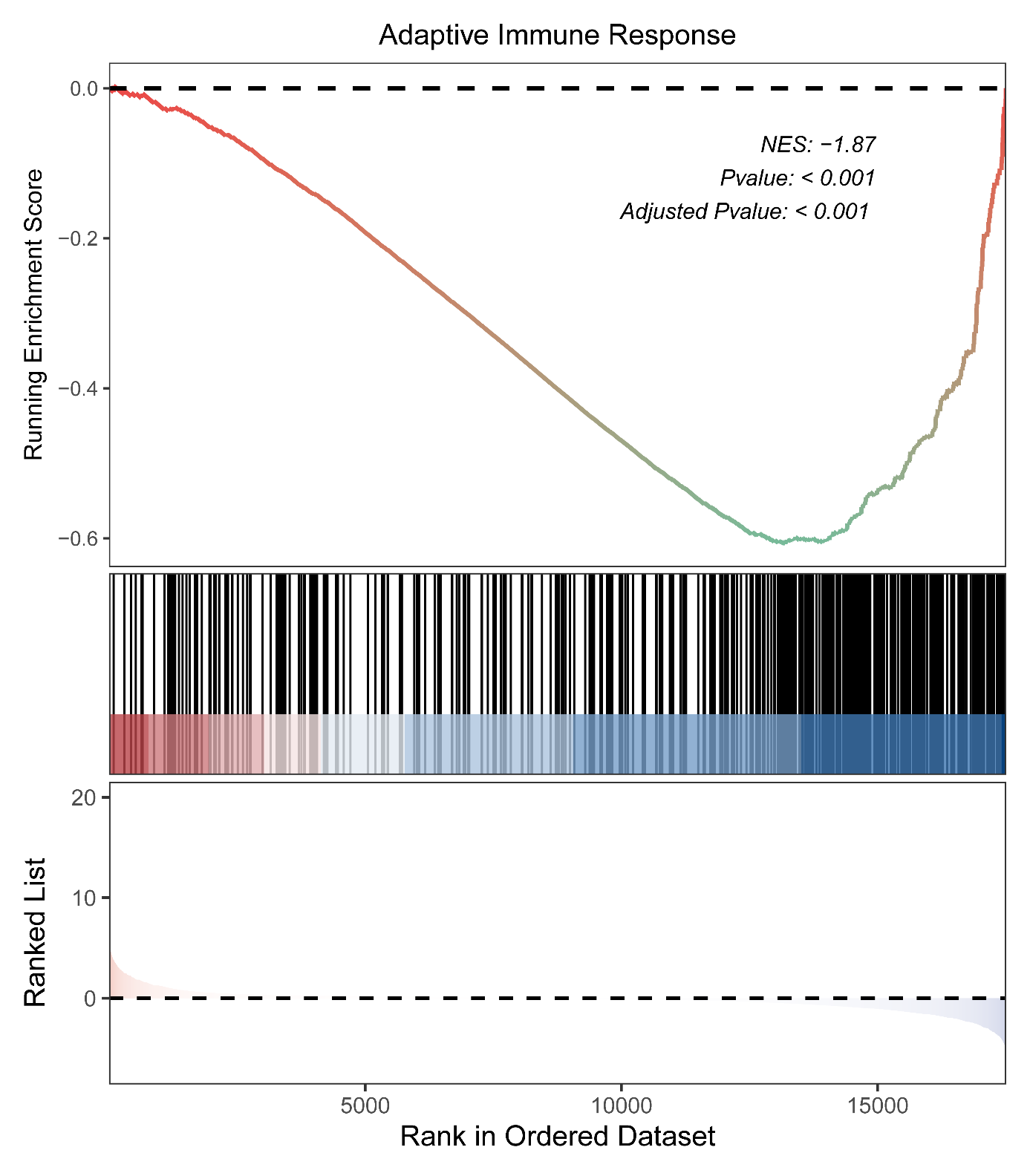


**Figure S24**: The significantly down-regulate2d enrichment gene sets by GSEA about the adaptive immune response through GO enrichment analysis (control vs *Fn*).


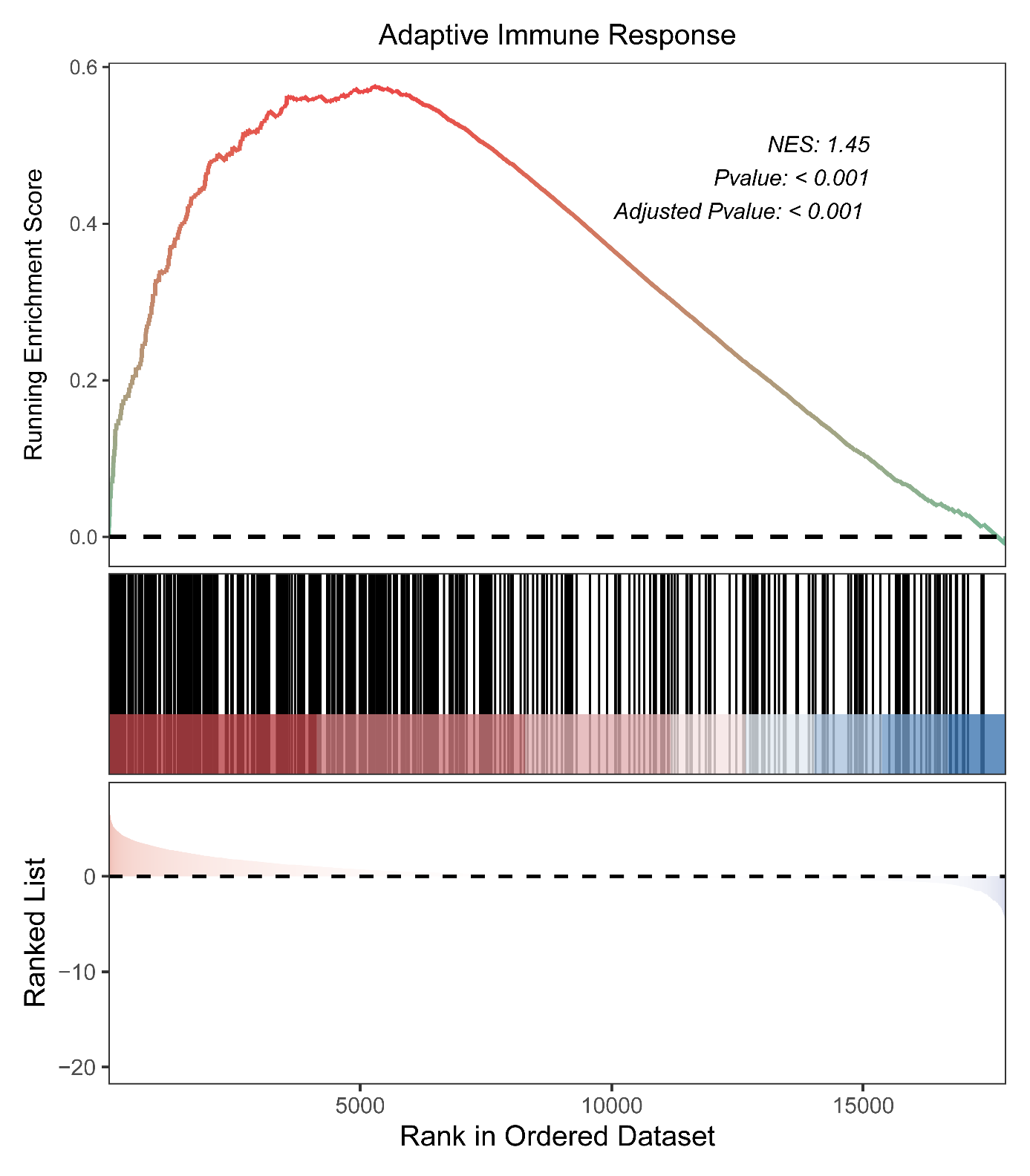


**Figure S25**: The significantly up-regulated enrichment gene sets by GSEA about the adaptive immune response through GO enrichment analysis (*Fn* vs M-MAP+US).


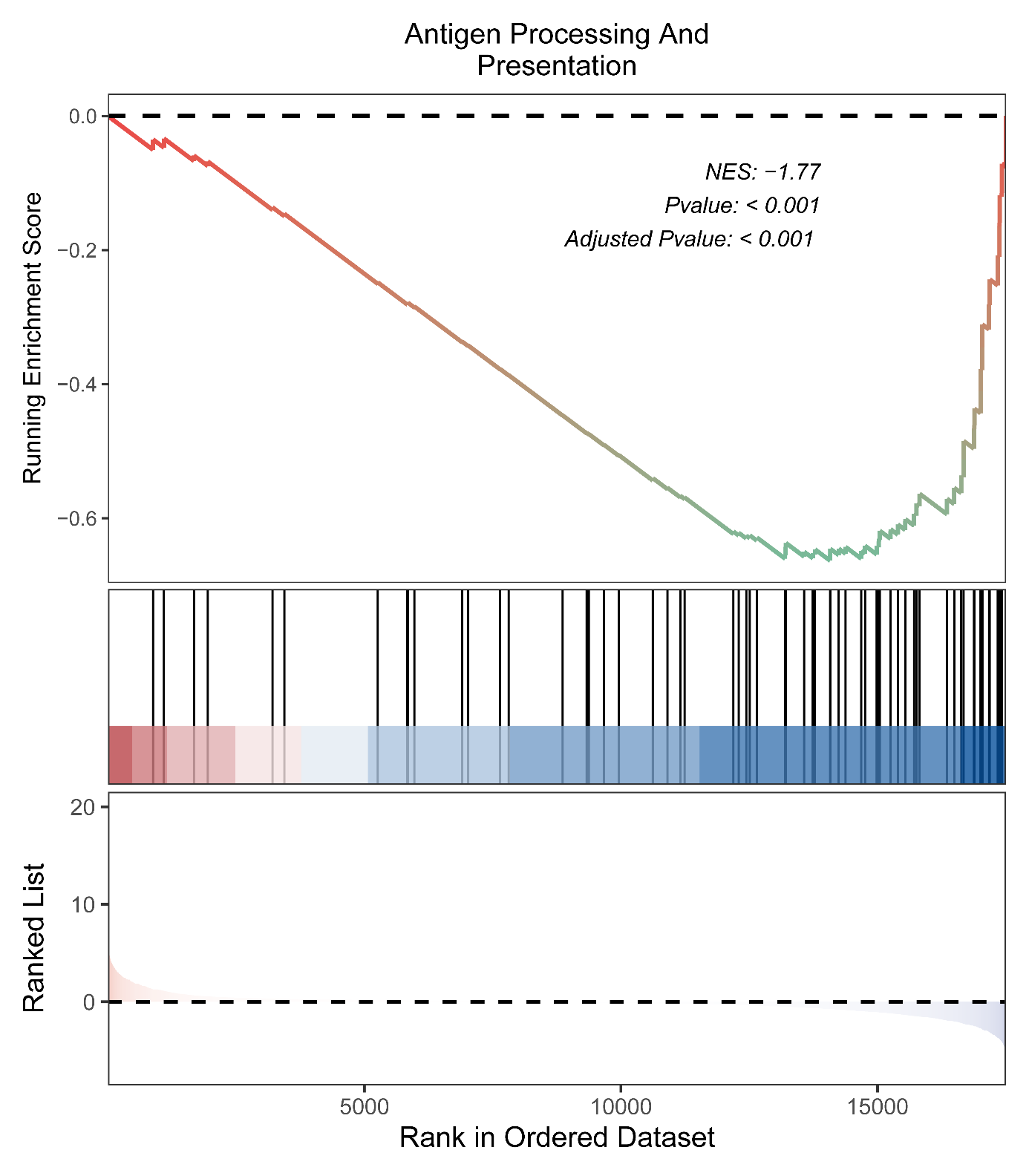


**Figure S26**: The significantly down-regulated enrichment gene sets by GSEA about the antigen processing and presentation through KEGG pathway enrichment analysis (control vs *Fn*).


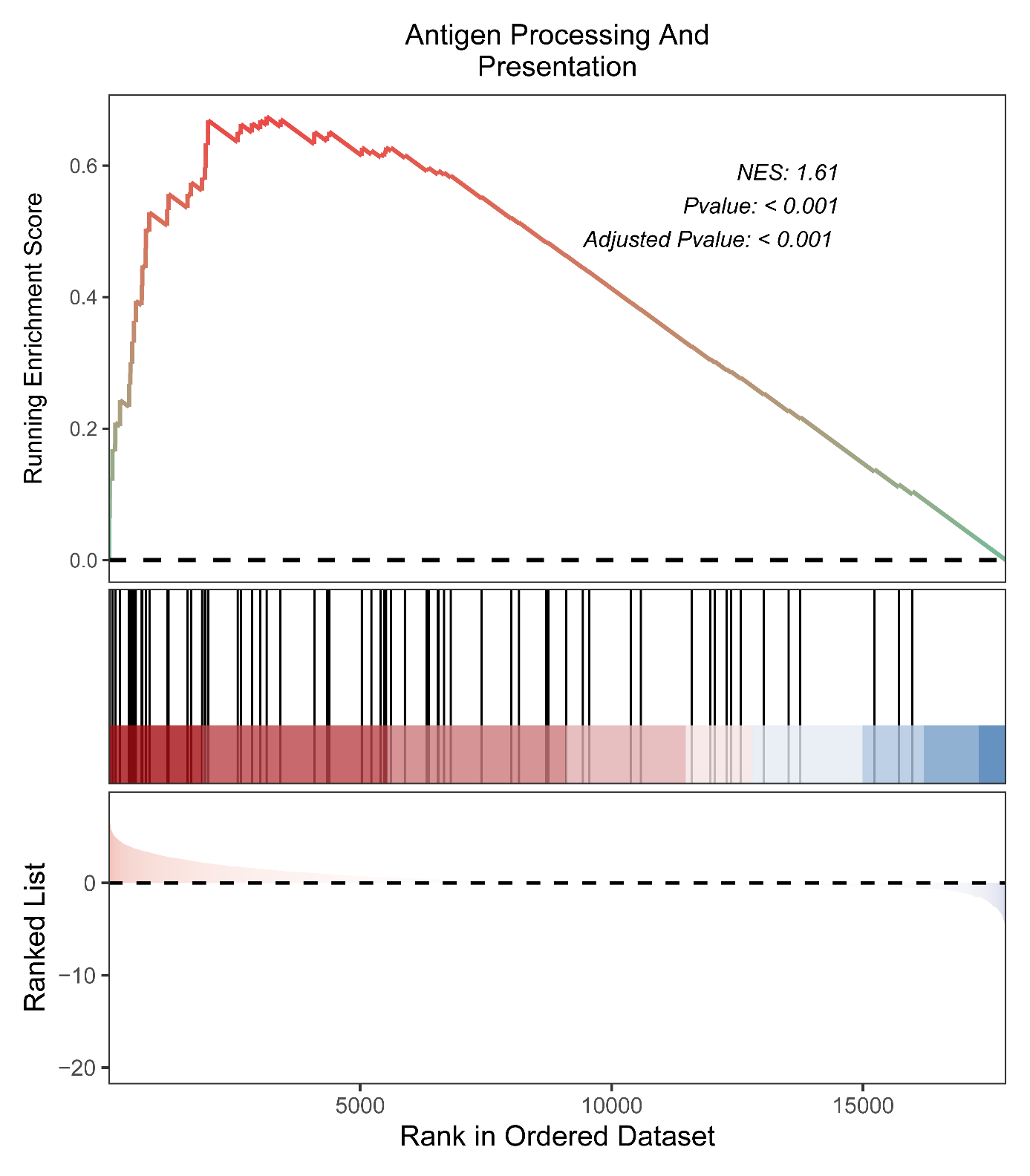


**Figure S27**: The significantly up-regulated enrichment gene sets by GSEA about the antigen processing and presentation through KEGG pathway enrichment analysis (*Fn* vs M-MAP+US).


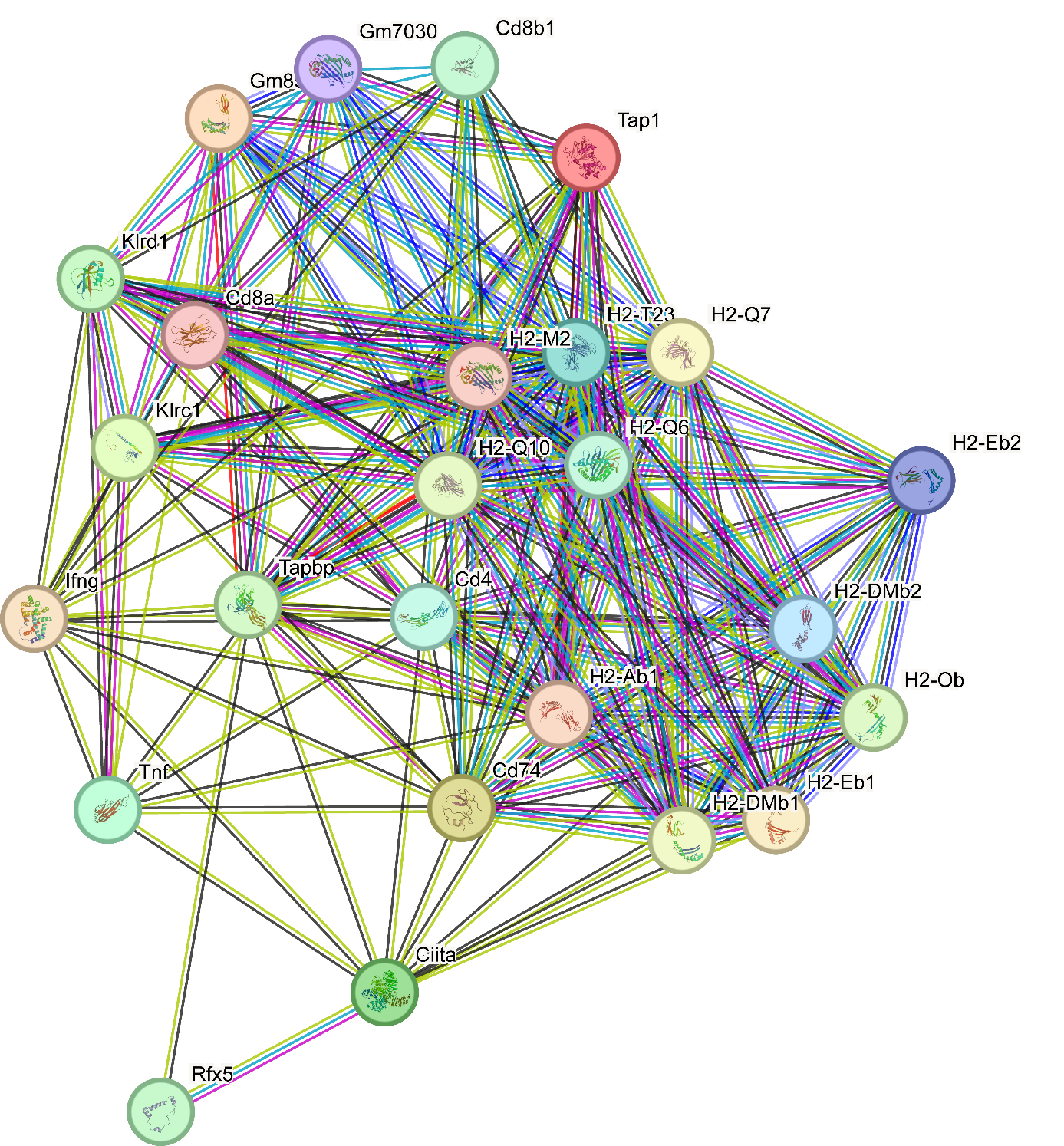


**Figure S28**: The protein–protein interaction analysis (PPI) network of the antigen processing and presentation pathway-related DEGs.


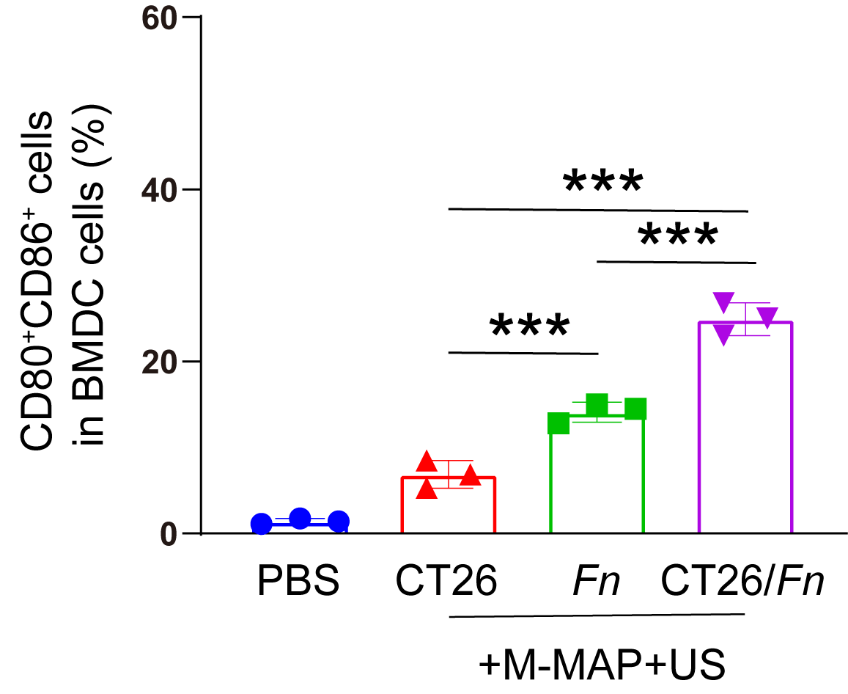


**Figure S29**: quantitative assessment of the amount of CD80^+^CD86^+^ cells in BMDCs based on flow cytometric results in b) (n=3). Data are presented as means ± SD. *** P < 0.001.


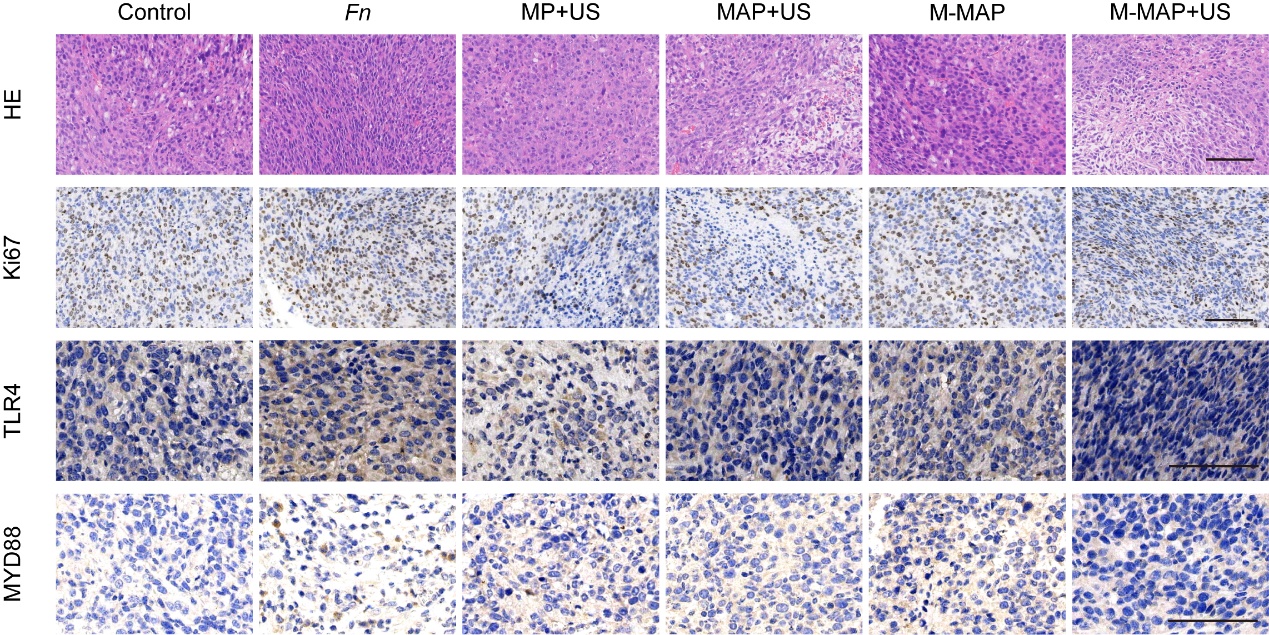


**Figure S30**: Representative images of H&E, Ki67, TLR4 and MYD88 staining of excised primary tumors of bilateral CT26 tumor model under different treatments (bar=100 µm).


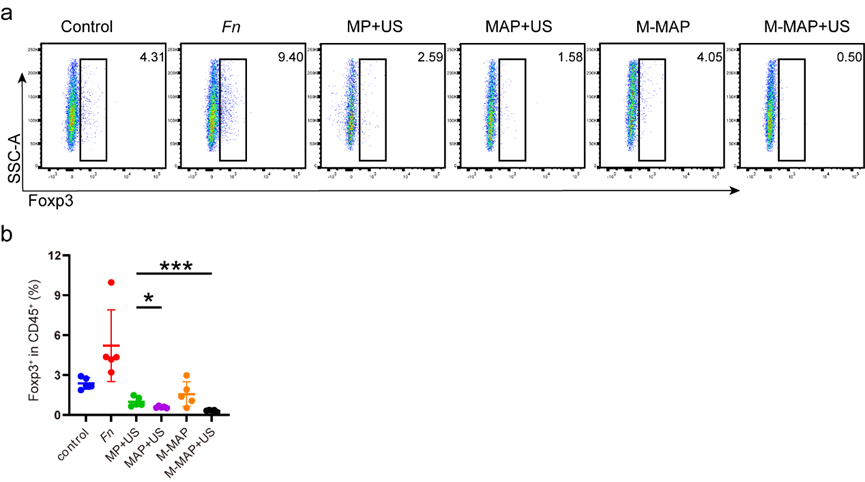


**Figure S31**: (a) The representative flow cytometric plots of Foxp3^+^ T cells in primary tumors from different experimental groups. (b) The quantification of Foxp3^+^ T cells in various groups (n=5). Data are presented as means ± SD. * P < 0.05, *** P < 0.001.


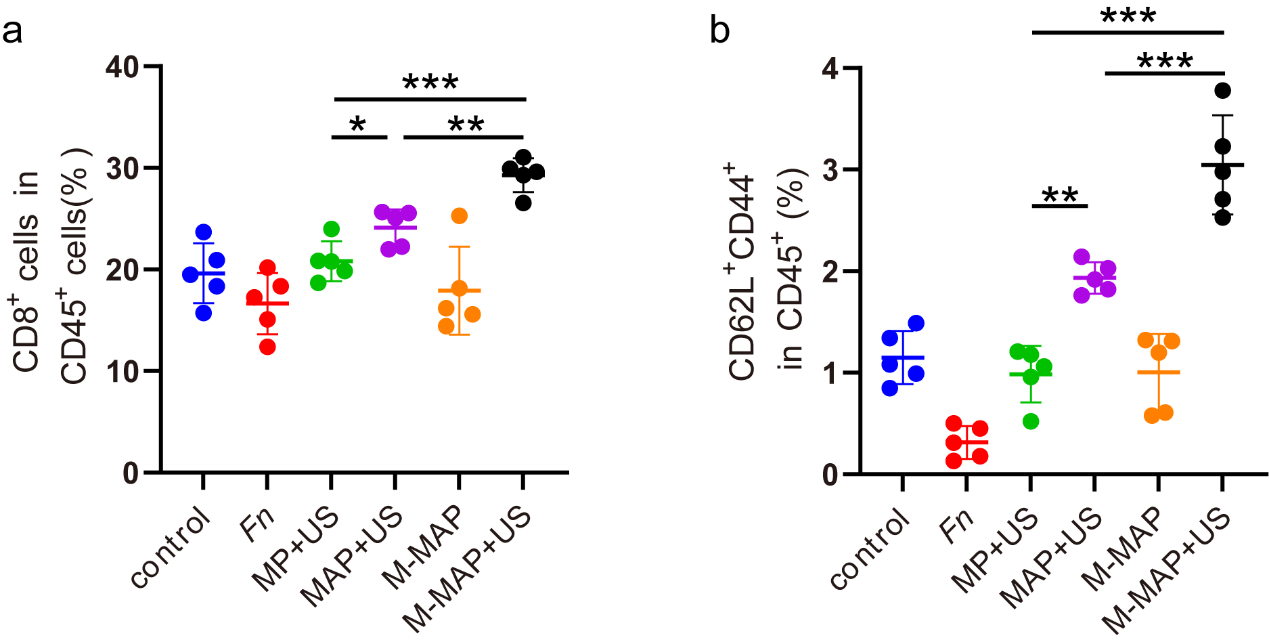


**Figure S32**: The quantification of CD8^+^ T cells (a) in distal tumor sites and central memory T cells (b) in spleens from different groups of bilateral CT26 tumor model (n=5). Data are presented as means ± SD. * P < 0.05, ** P < 0.01, *** P < 0.001.


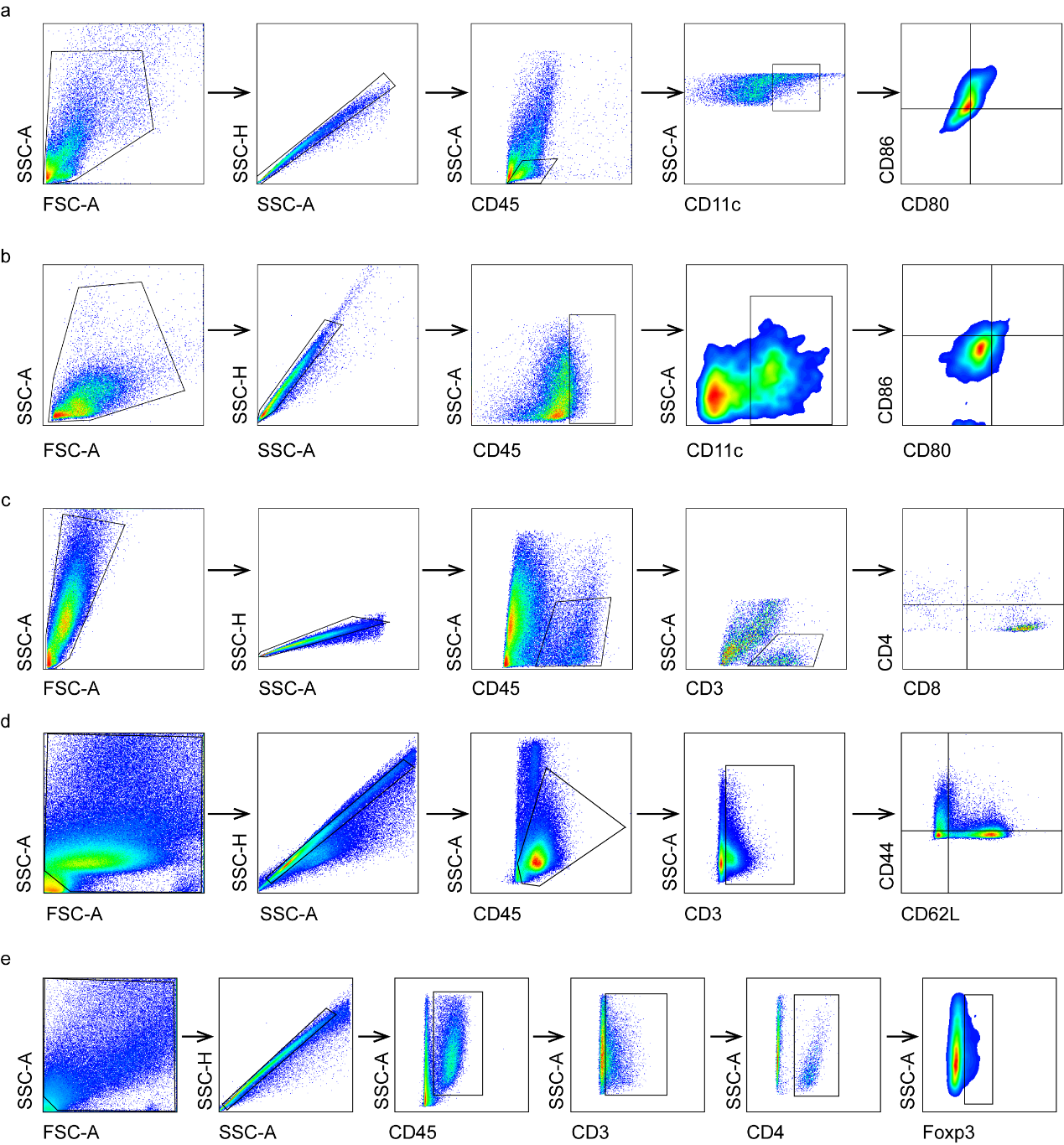


**Figure S33**: Gating strategies used for flow cytometry analysis of immune cells in this study. (a) Gating strategy to analyze CD80^+^CD86^+^ cells in BMDC cells. (b) Gating strategy to analyze matured DC cells (CD80^+^CD86^+^) from the spleen in CT26-luc/*Fn* orthotopic tumor mouse model. (c) Gating strategy to analyze CD45^+^CD3^+^CD4^+^ or CD45^+^ CD3^+^CD8^+^ T cells from tumor in CT26-luc/*Fn* orthotopic tumor mouse model and bilateral CT26 tumor model. (d) Gating strategy to analyze CD44^+^ CD62L^+^ T central memory cells from spleen in bilateral CT26 tumor mouse model. (e) Gating strategy to analyze Treg cells in bilateral CT26/*Fn* tumor mouse model.


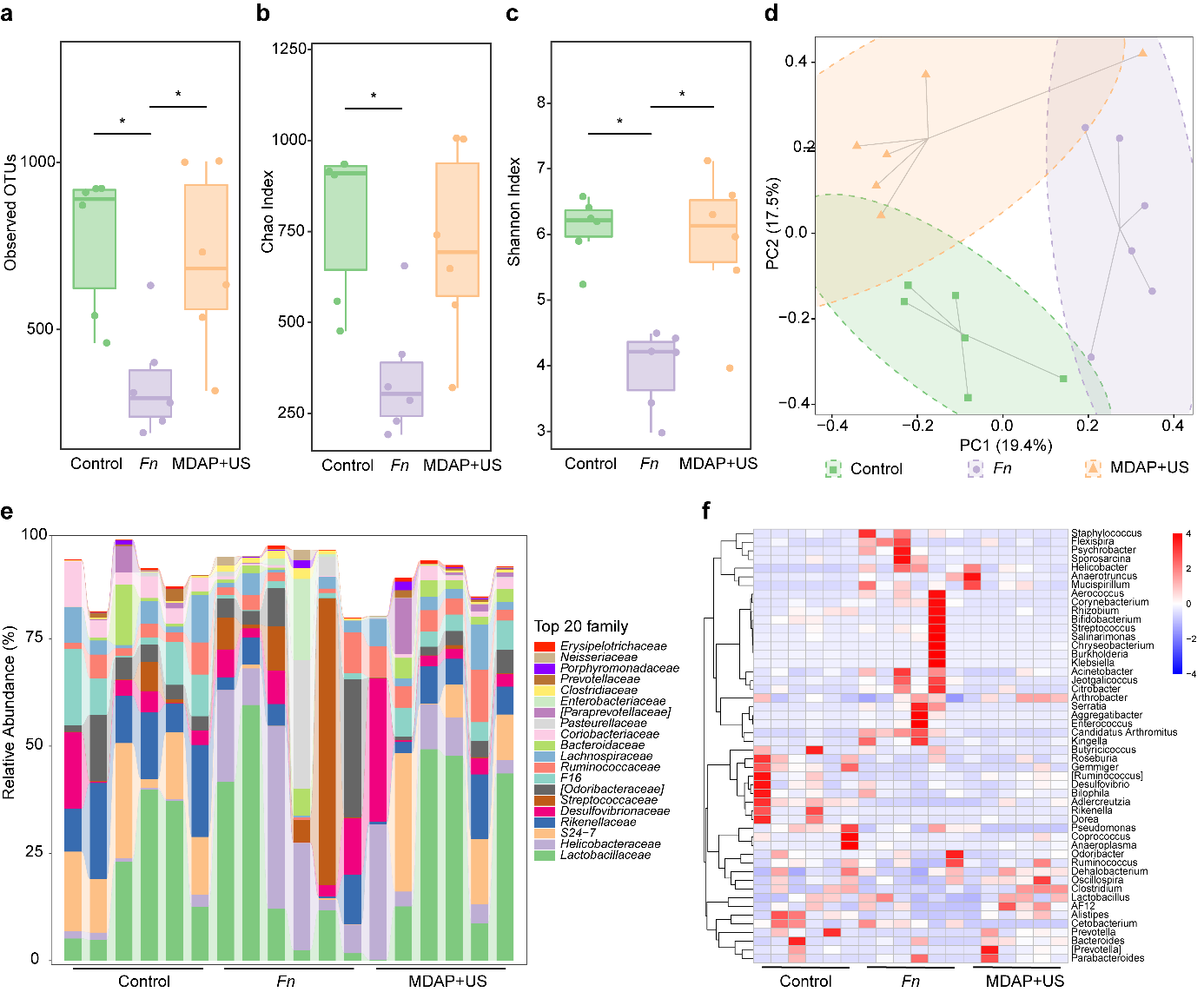


**Figure S34**: Injecting M-MAP with SDT intravenously helped maintain the balance of intestinal flora in mice. (a) Estimated richness of microbial communities (observed OUTs). b, c) Alpha diversity evaluated by Chao (b) and Shannon (c) indices. (d) PLS-DA plots of beta diversity illustrating microbial compositions. (e) Taxonomic bacterial distribution histogram by family. (f) Euclidean distance-based clustered heatmap at the genus level (n=6).

**Table S1**: Primer sequences used in this study.

| **Gene** | **Primer sequences (5’-3’)** |
| --- | --- |
| *Fn* | F: CTTAGGAATGAGACAGAGATG |
|  | R: TGATGGTAACATACGAAAGG |
| GAPDH | F: AGGTCGGTGTGAACGGATTTG |
|  | R: TGTAGACCATGTAGTTGAGGTCA |

**Reference**

[1] Q. Chen, T. Liu, S. Chen, Y. Luo, M. Ma, F. Xue, L. Zhang, W. Bao, H. Chen, *ACS Appl Mater Interfaces* **2019**, 11, 45404.

[2] Y. Yang, Y. Zhu, K. Wang, Y. Miao, Y. Zhang, J. Gao, H. Qin, Y. Zhang, *Bioact Mater* **2023**, 29, 116.

[3] M. Kanehisa, S. Goto, *Nucleic Acids Res* **2000**, 28, 27.
